# Expert Evaluation and Consensus on GPT-4o Summaries of Clinical Letters: Validation and Results of the Framework and Implementation of AI Tools Project

**DOI:** 10.2196/90374

**Published:** 2026-05-11

**Authors:** Mieke Deschepper, Helga Rogge, Kirsten Colpaert

**Affiliations:** 1 Data Science Institute, Ghent University Hospital, Corneel Heymanslaan 10, Ghent, Flanders, 9000, Belgium, +32 9 3321814; 2 Department of Internal Medicine and Pediatrics, Ghent University, Ghent, Flanders, Belgium

**Keywords:** large language model, LLM, clinical letters, expert agreement, interrater reliability, hallucinations, omissions, consensus, natural language processing, artificial intelligence, health care AI

## Abstract

**Background:**

Large language models (LLMs) are increasingly used to summarize clinical documents; yet, automated metrics often inadequately capture clinical relevance and safety. In the initial phase of the “Framework and Implementation of AI Tools,” an expert-driven, cocreated evaluation methodology was established to assess LLM-generated discharge letter summaries, combining prompt design considerations with intuitive expert appraisal.

**Objective:**

This study aimed to quantify expert agreement and interrater reliability on LLM summaries of discharge letters, identify frequent and clinically relevant errors, and evaluate practical implications for integrating LLMs into documentation workflows.

**Methods:**

Thirty expert-curated synthetic Dutch discharge letters were summarized. Thirty-one clinicians from Flemish care settings (1 university hospital, 2 private hospitals, and 2 general practice circles) evaluated the summaries. The evaluation framework consisted of 61 binary layout items assessing whether required sections and formatting were correctly present, 33 content items (correct or complete vs incorrect, subcategorizing missing, irrelevant, and hallucinated information), a 4-point global quality rating, and an open comment. Statistical analyses included descriptive statistics, mixed effects ordinal regression on the global score, consensus (agreement per question or letter) percentages, interrater reliability (Cohen κ, intraclass correlation coefficient [ICC], Fleiss κ, and prevalence index), and thematic synthesis of comments.

**Results:**

Layout adherence was high (88%), especially in the conclusion section. The positive response rate for content was overall moderate (78%), with the best performance observed in the medical history section and the lowest performance observed in the medication section, which also showed the highest rate of hallucinations and the weakest interrater consensus. Across all sections, missing information was the most common error. Nearly 70% of global ratings were “good” or “very good.” Higher positive response rates for content predicted better global scores (β=.079; *P*<.001), while layout and participant specialty were not relevant to global scoring. Consensus was high for the layout questions (median 96.8%, IQR 90.2%-100%) and somewhat lower for content (median 83.9%, IQR 67.7-96.8), with the lowest agreement in the medication section. Interrater agreement was moderate (median Cohen κ=0.36, IQR 0.29-0.43; range 0.07‐0.56), but overall reliability was high (ICC 0.945, 95% CI 0.942-0.948), indicating strong consistency at the global level despite interrater variability. The prevalence index demonstrated that high ICC values were partly driven by the strong prevalence of affirmative responses in layout items, while content items showed more balanced distributions and lower agreement.

**Conclusions:**

Our framework offers a robust approach for evaluating LLM-generated discharge summaries, balancing usability and clinical relevance. Semantic integrity, especially regarding medication details, was identified as a key vulnerability. Perceived overall quality was driven by a positive response rate for content. High ICC values for global score, with lower item-level agreement lead toward the need for clearer, context-specific prompts and standardized evaluation criteria to reduce interrater variability. Human oversight and targeted automated checks for omissions and hallucinations are essential for safe clinical deployment.

## Introduction

The evaluation of large language models (LLMs) in health care has predominantly centered on accuracy metrics; yet, only a small fraction of studies (6.45%) specifically address the evaluation of medical LLMs [1]. Recognizing the limitations of this narrow focus, the “Framework and Implementation of AI Tools” (FRAIT) project [2] established a collaborative, cocreative workflow with hospital providers to develop a more comprehensive evaluation approach. Building on the structured planning principles outlined by Tam et al [3], the FRAIT project emphasized the importance of preparatory phases before human evaluation.

Unlike prior studies that often relied on general or model-centric questions [4], our approach prioritized trustworthiness and a detailed examination of individual sections within medical summaries. A key innovation was the separation of evaluation criteria into “layout” (the presence and structure of required sections) and “content” (the accuracy and completeness of clinical information). This distinction acknowledges that while layout can be objectively assessed, content evaluation is inherently more complex and nuanced.

Recent literature [5] underscores the necessity of using real-world patient care data to ensure that LLM evaluations reflect actual clinical conditions. There is a growing consensus on the need for standardized frameworks that define evaluation tasks and dimensions, address specialty-specific gaps, and mitigate potential biases. Additionally, comprehensive cost-benefit analyses and transparent reporting of AI system failures are essential for the responsible integration of LLMs into clinical workflows.

Traditional automated metrics such as ROUGE and BLEU, although widely used, have been shown to correlate poorly with expert human assessments in clinical contexts [6,7]. These metrics often overlook critical aspects such as semantic accuracy, clinical relevance, and contextual appropriateness. Consequently, expert-driven, consensus-based evaluation frameworks are increasingly advocated.

LLM-powered summarization tools hold significant promise for distilling essential information from lengthy discharge letters and medical records, thereby supporting clinicians in making timely, informed decisions [8]. Reliable summarization methods can enhance workflow efficiency, reduce cognitive burden, and improve care quality. However, the adoption of LLMs in clinical documentation also raises concerns regarding accuracy, safety, and interpretability.

To address these challenges, the FRAIT project systematically evaluated LLM-generated summaries of expert-curated synthetic letters using a diverse panel of health care professionals. An intuitive evaluation tool was developed and applied to medical discharge letters, enabling a thorough analysis of both structural and semantic aspects.

The aim of this study was to systematically evaluate the reliability, accuracy, and usability of LLM-generated summaries of clinical discharge letters using the consensus-based FRAIT framework. By engaging a multidisciplinary panel and using a structured evaluation tool, we sought to (1) quantify expert agreement on summary quality, (2) identify the most frequent and clinically relevant errors, and (3) assess the practical implications of integrating LLMs into clinical documentation workflows.

## Methods

We followed the QUEST (Quality of Information, Understanding and Reasoning, Expression Style and Persona, Safety and Harm, and Trust and Confidence) human evaluation framework [3] to set up our method in Appendix 1.1 in Multimedia Appendix 1.

### Participants

A total of 33 clinicians (including 2 nonphysicians) were recruited from 3 hospitals (University Hospital Ghent, AZ Sint-Lucas Ghent, and AZ Oudenaarde) and 2 general practitioner circles (Huisartsenkring Schelde en Leie and Huisartsenvereniging Ghent). Two physicians did not complete the evaluation due to technical login issues (n=1) or time constraints (n=1). Half of the group (n=16, 51.6%) were hospital-based specialists, followed by general practitioners (n=13, 42%), with the remainder consisting of other health care professionals (n=2, 6.5%; Table 1). All participants evaluated 30 summaries from the respective medical discharge letters. More details on the number of years of clinical practice for each participant type can be found in Appendix 1.2 in Multimedia Appendix 1.

**Table 1.** Overview of organization and participants.

Type of organization (n=31) and name	Values, n (%)
Hospital (n=19, 61.29%)	
AZ Oudenaarde	5 (16.13)
AZ Sint-Lucas Ghent	6 (19.35)
Ghent University Hospital	8 (25.81)
GP^a^ circles (n=12, 38.71%)	
Huisartsenvereniging Ghent	10 (32.26)
Huisartsenkring Schelde en Leie	2 (6.45)

a GP: general practitioner.

### Workshop Design

A workshop on user interface and user experience design was conducted on October 23, 2024, at Ghent University Hospital. The primary objective was to explore how an evaluation tool for LLM-generated summaries should be structured from a physician’s perspective. A total of 26 participants attended, representing all participating organizations. Feedback collected during the workshop was synthesized and shared with technical implementers to finalize the design of the evaluation tool. The user interface was tested by 2 individuals, one data scientist (MD) and one physician (HR). A subsequent test session involved 5 additional technical participants and 3 physicians. These sessions focused on usability, clarity, and alignment with clinical workflows.

### Evaluation Timeline

**Figure 1.**
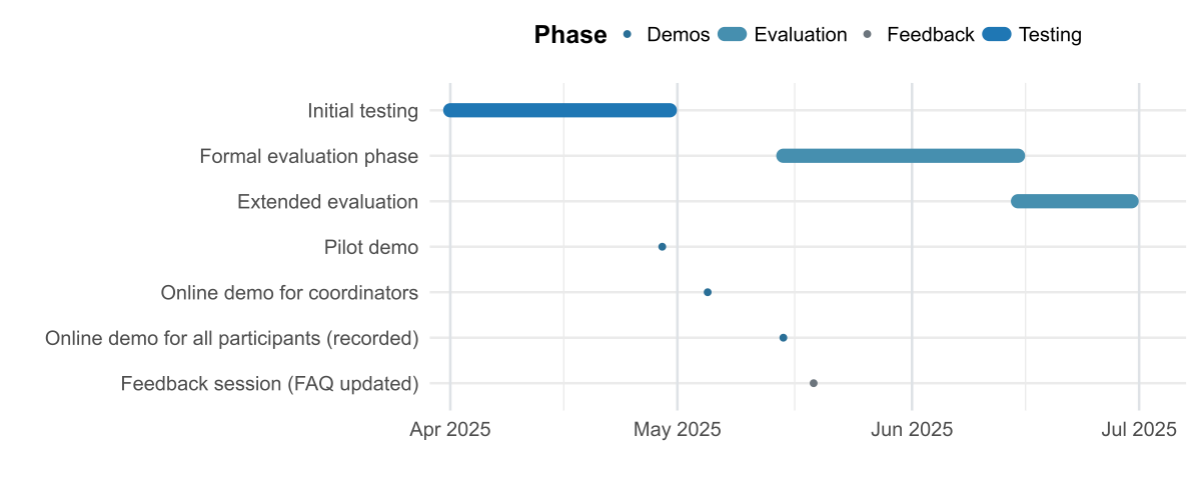
Evaluation timeline on the Framework and Implementation of AI Tools (FRAIT) project. Starting from April 1, 2025, with the initial testing until the demonstration of the tool at the FRAIT symposium on October 22, 2025. FAQ: frequently asked question.

The evaluation phase timeline is illustrated in Figure 1. A total duration of 6 weeks was allocated for the completion of the evaluation. From April 1 to April 30, 2025, initial testing of the evaluation tool was conducted by 2 physicians and 1 data scientist. A pilot demonstration was held for the Data Science Institute from Ghent University Hospital on April 29, 2025. Following feedback from this session, a slide-based walkthrough was developed to clarify procedural steps prior to demonstrating the tool. An online presentation for (local) coordinators at participating institutions took place on May 5, 2025; participants received a manual with screenshots and keyboard shortcuts. All coordinators evaluated the tool over a week and reported no major issues.

A broader online demonstration for all participants occurred on May 15, 2025, and was announced via newsletter and email. This session included login instructions for the secure platform and a comprehensive walkthrough of the evaluation tool. For those unable to attend, a recording of the session was made available. On May 19, 2025, a follow-up feedback session addressed questions, which were subsequently compiled into a frequently asked questions document distributed to all users. The formal evaluation phase spanned from May 15 to June 15, 2025, with an extended period continuing until June 30, 2025. Subsequently, a brief demonstration was presented by a general practitioner during the FRAIT symposium on October 22, 2025.

Several educational resources were provided for physicians, including an extensive manual with sections such as examples of potential questions, a live demonstration with an available recording, and a dedicated question-and-answer session. Additionally, during the evaluation phase, support was offered via email and telephone during working hours.

### Input for the Evaluation Tool

The evaluation was conducted using a standardized prompt derived from the FRAIT questionnaire [2]. This prompt was designed to capture essential clinical and structural elements of discharge summaries across predefined categories: *general, medical history, investigations, hospital course, medication, follow-up,* and *conclusion*. An overview of the number of questions per category is provided in Appendix 2.1 Multimedia Appendix 2.

### Data and Materials

Thirty expert-curated synthetic Dutch medical discharge letters were generated according to the protocol outlined in Appendix 3 in Multimedia Appendix 3. For the purpose of this study, we used the term synthetic clinical letters to describe synthetically modified clinical documents derived from real patient records that were systematically transformed to prevent reidentification while preserving clinical structure and plausibility. This process included complete removal of all direct and indirect personal identifiers, temporal shifting of dates, and targeted modification of clinical content (eg, laterality, diagnoses not relevant to the clinical scenario, and physiological and laboratory parameters) where deemed necessary. To minimize the risk of medical logic drift introduced by shifting dates, all synthetic letters were manually generated by 1 physician and independently reviewed by a senior physician to ensure that clinical decisions, treatments, and guideline-based care remained historically plausible despite the temporal adjustments. The resulting letters do not correspond to any real individual but retain realistic clinical narratives suitable for evaluation purposes. All data were processed to eliminate any risk of patient reidentification, and the approach was reviewed and approved by the relevant institutional committees (reference ONZ-2024‐0273 and ONZ-2024‐0304). These letters served as the source material for summarization and subsequent evaluation. The LLM used for this task was GPT-4o, configured with a temperature setting of 0 to minimize interpretative variability. In this phase, one single prompt was used to be able to evaluate the same results.

### Evaluation Framework

**Figure 2.**
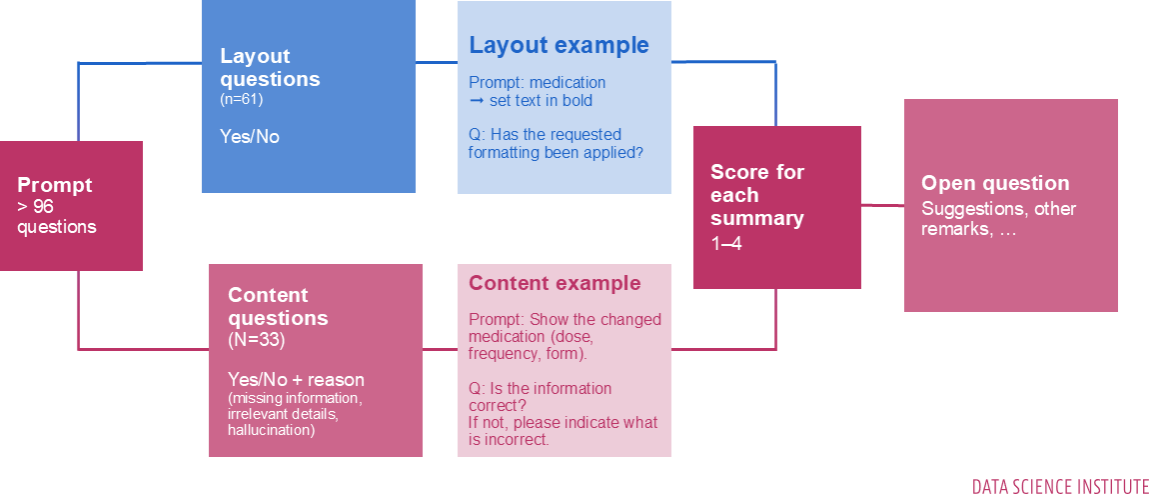
Overview of the 96 evaluation questions: layout, content, global score, and open question. A full overview of all questions can be found in Appendix 2.2 in Multimedia Appendix 2.

The evaluation questions were organized into 4 categories (Figure 2). Layout questions (n=61) were binary and assessed whether the specific sections requested in the prompt were present in the generated summary (for instance, evaluators were asked: *“Show the General section in the summary. Is the requested section included?”*). Additionally, these questions assessed the format in which sections should be presented, such as bullet points or plain text. Content questions (n=33) examined the correctness and completeness of individual items. Responses could be *yes* or *no*, with the latter requiring further classification as *incomplete*, *too much or irrelevant information*, or *incorrect information or hallucination*. When answering *no*, evaluators were required to indicate the erroneous content in either the source or the summary. An example question was *“Show the Admission Date. Is the content correct? If not, indicate what is incorrect.”* In addition, the evaluation framework comprised 4 distinct categories of items: layout, content, global scoring, and an open question. Layout items assessed compliance with prompt-specified structural requirements and were scored as binary (yes or no). For each layout item, participants were able to view the original prompt and asked to evaluate whether the LLM-generated summary adhered exactly to the requested formatting and structure (eg, presence of specific section headings, use of bold formatting, bullet lists with a maximum number of items, or mandated section titles such as “medication”). Strict adherence was required for layout items to reflect real-world prompt-based use of LLMs in clinical documentation workflows.

Content items evaluated the correctness and completeness of the generated summaries relative to the original clinical text and were also scored binary (yes or no). When content was rated as incorrect (no), participants were required to assign one or more predefined error categories: missing information (clinically relevant information present in the source text but absent from the summary), irrelevant details (information present in the source text but not requested or inappropriate for the section, including excessive or misplaced detail), or hallucinations (information not present in the original clinical text).

The global score question (n=1) asked evaluators to provide a global rating of the summary quality on a 4-point scale: *very bad, bad, good,* and *very good*. Finally, an open question (n=1) invited evaluators to add comments, provide suggestions, or note blind spots in the original letter that were not captured by the prompt.

This structured approach ensured a comprehensive assessment of both structural fidelity and semantic accuracy. Figure 2 provides an overview of the evaluation question types. The set of evaluation questionnaire, including all layout and content items, is provided in Appendix 2.2 in Multimedia Appendix 2.

### Statistical Analysis

#### General Approach

Descriptive statistics were computed to characterize participants, organizational affiliation, and medical specialty, expressed as frequencies (n) and percentages. In addition, descriptive analyses summarized the overall performance on layout and content questions, both globally and stratified by category.

#### Score Analysis

The global score question offered 4 ordinal response options: *very bad* (1), *bad* (2), *good* (3)*,* and *very good* (4). We deliberately selected a 4-point Likert scale without a neutral midpoint to avoid noncommittal responses and to encourage evaluators to make a clear judgment on the overall quality of each summary. These were reported as frequencies and percentages and visualized using heatmaps by participant and by discharge letter. To explore associations between summary quality and evaluation metrics, a mixed effects ordinal regression model was applied. The dependent variable was the global score (treated as ordinal, 1‐4), while predictors included the number of “yes” responses for content and layout questions (analyzed separately) and participant specialty. Participant identity was modeled as a random effect to account for intrarater correlation.

#### Consensus Assessment

For each question pertaining to the same discharge letter, agreement among participants was examined. Responses were dichotomized as yes or no*,* without distinguishing the reason for a negative response in content-related items. Disagreement within a question-letter pair was recorded, and consensus was aggregated across dimensions (content and layout) and categories.

#### Interrater Reliability

To evaluate consistency among raters, multiple measures of interrater agreement were used. Pairwise agreement was quantified using Cohen κ for all rater pairs across content and layout questions. The median κ values per rater were compared using the Kruskal-Wallis test, followed by Dunn post hoc test when significant differences were observed. The number and proportion of significant differences were reported (n, %). The intraclass correlation coefficient (ICC) was calculated for all questions combined (content treated as binary: yes or no) and for the global score assigned to each summary. ICC was recalculated after excluding outlier raters to assess robustness. For multirater agreement on content questions, Fleiss κ was computed, both overall and by category. As Fleiss κ lacks a standard CI, a 95% CI was estimated via bootstrapping (10,000 iterations). Results were visualized using heatmaps and box-violin plots for pairwise Cohen κ and point plots with error bars for ICC and Fleiss κ estimates.

To better understand discrepancies between agreement metrics, we calculated the prevalence index (PI) for all binary items. The PI measures how strongly responses lean toward “yes” or “no,” ranging from 0 (balanced) to 1 (highly skewed). High prevalence can lower Cohen κ and inflate ICC because ICC is influenced by between-item variance rather than categorical balance. We therefore computed the PI both overall and per category for layout and content items to assess how prevalence contributed to differences between κ and ICC. We also calculated the mean PI per category to capture item-level skewness. While the overall PI reflects global imbalance across the dataset, the mean PI shows how consistently individual items display skewed response patterns. Reporting both helps distinguish whether prevalence effects stem from a few extreme items or from systematic imbalance across categories.

### Sensitivity Analysis

A sensitivity analysis was performed comparing results from all raters with those obtained exclusively from the physician cohort. Further details can be found in Multimedia Appendix 4.

### Qualitative Analysis

Responses to the open-ended question were analyzed manually and summarized thematically to identify recurring topics and suggestions.

### Ethical Considerations

This study involved the evaluation of LLM-generated summaries of *synthetically modified* discharge letters. All procedures complied with institutional and national regulations governing the secondary use of clinical data for research.

1. Human subjects research ethics review, exemptions, and approvals: The protocol for generating and using synthetic clinical discharge letters was reviewed and approved by the Ethical Committee of Ghent University Hospital (reference ONZ20240273 and ONZ20240304). The Ethical Committees of AZ Sint-Lucas Ghent and AZ Oudenaarde were informed of the study in accordance with institutional governance requirements. The Committee determined that the study met applicable national and institutional regulatory requirements governing secondary use of health data. The institutional Data Protection Officer (DPO) was formally consulted during protocol preparation. The DPO reviewed the planned data flow, synthetic transformation procedures, and external private cloud deployment. Given that only synthetically transformed data were processed externally, the DPO and Ethics Committee determined that the study did not meet the institutional threshold for a full Data Protection Impact Assessment. The residual privacy risk was classified as low and proportionate to the research objectives.
2. Informed consent or waiver language: The source documents used to generate the synthetic letters originated from previously collected hospital records of deceased patients. For this retrospective secondary use, the Ethics Committee granted approval and waived the requirement for additional patient consent. The Committee determined that, given the exclusive inclusion of deceased patients, the retrospective design, and the implementation of structured deidentification and synthetic transformation procedures prior to further processing, the study did not require renewed consent. Although no anonymization method can guarantee absolute elimination of risk, all direct identifiers were removed, and the transformation process was designed to minimize any potential risk of reidentification.
3. Privacy and confidentiality protection: All clinical discharge letters underwent a rigorous structured deidentification and synthetic transformation procedure by protocol before use in the evaluation tool, including the removal of direct identifiers and the generalization or modification of indirect identifiers, temporal shifting of dates, modification of clinical elements when necessary to reduce reidentification risk, and internal review of transformed letters to assess residual linkage risk. These procedures were designed to preserve clinical realism while minimizing the likelihood of reidentification. Although no anonymization strategy can fully eliminate risk, the applied safeguards were considered proportionate to the study objectives and were reviewed as part of the approved protocol. The evaluation was conducted within a secure, access-controlled hospital environment with authenticated access and governance in accordance with institutional data protection standards.
4. Compensation: The study was supported by governmental research funding. Participating institutions received financial support to facilitate clinician participation in accordance with local institutional policies. Compensation arrangements were managed at the institutional level and reflected time commitment only; they were not dependent on performance or evaluation outcomes.
5. Protection of participant identity in figures and supplementary material: All clinician participants were assigned anonymized alphanumeric codes (eg, “Z19”) for all analyses and visualizations. Figures and appendices have been updated to replace any synthetic first names with anonymous codes. No identifiable images or personal information of participants are included in the manuscript or supplementary material. If identifiable material were to be included in future dissemination, explicit written consent would be obtained and submitted as required.

## Results

### Evaluation Tool

**Figure 3.**
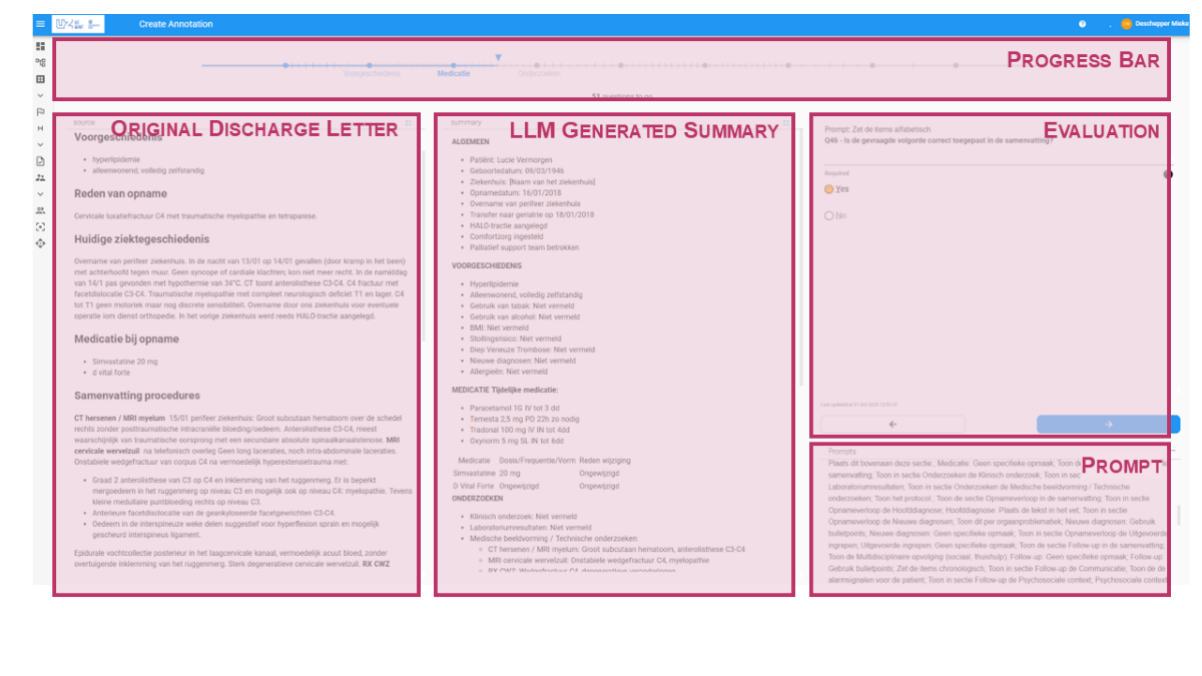
Screenshot of the evaluation tool highlighting its main components.

The workshop generated numerous suggestions for enhancing the FRAIT evaluation tool. A comprehensive list of implemented features is provided in Appendix 5.1 in Multimedia Appendix 5. Examples include keyboard shortcuts for navigation, progress indicators, autosave functionality, and text highlighting. Figure 3 illustrates the main components of the evaluation interface, while additional screenshots and examples of the content question user interface are included in Multimedia Appendix 5.

### Evaluation Outcomes

Across all layout-related questions, 88% of responses were rated as “yes,” with the highest proportion observed in the *conclusion* section. For content-related questions, 78% of responses were affirmative, with the *medical history* category achieving the highest score. In contrast, the *medication* section demonstrated the lowest positive response rate and the highest proportion of hallucinations. Overall, hallucinations accounted for 3% of content-related responses. When content questions were answered “no,” missing information was the predominant reason, particularly in the *conclusion* section. The *general* section contained the largest proportion of irrelevant details (Table 2).

**Table 2.** Percentage of answers divided by content and layout and by category.

Category	Domain: content (%)				Domain: layout (%)
	Yes (total=77.5)	No missing information (total=15.6)	No irrelevant details (total=4.8)	No hallucination (total=3.1)	Yes (total=88.4)
General	72.8	3.4	20.1	3.8	90.3
Medical history	83.7	12.6	1.8	1.9	83.8
Investigations	74.1	22.9	2.1	0.9	94.3
Hospital course	81.1	12.8	4.4	1.7	93.2
Medication	67.8	18.1	6.3	7.8	83.2
Follow-up	80	11.2	3.6	5.2	87.7
Conclusion	68.4	25.9	4.6	1.2	97.8

### Global Scores

**Figure 4.**
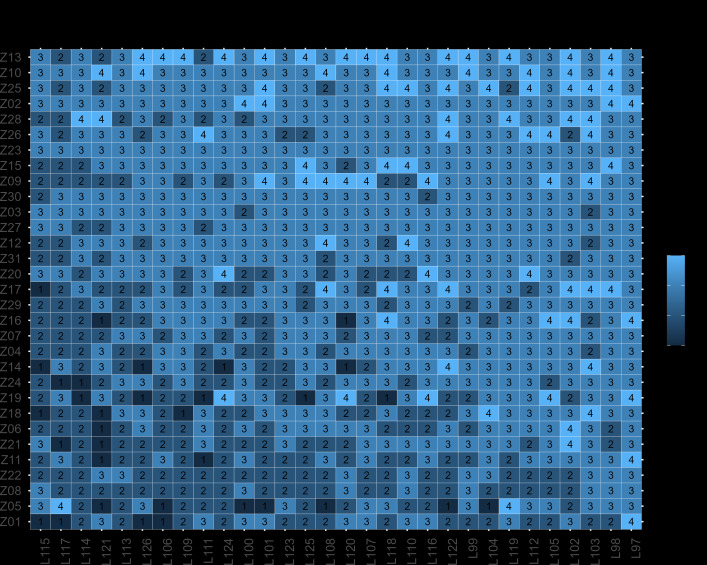
Heatmap of the global scores by participant (y-axis) and generated summary (x-axis).

Nearly 70% of participants rated summaries as *good* or *very good*. A detailed visual overview is presented in Appendix 6 in Multimedia Appendix 6. Figure 4 displays an ordered heatmap of global scores (1-4) by participant and discharge letter.

Mixed effect ordinal regression results are summarized in Table 3. The proportion of “yes” responses for content questions was significantly associated with higher global scores (β=.079; *P*<.001), whereas layout performance showed no significant effect. Participant specialty did not exhibit a significant influence on scoring.

**Table 3.** Mixed effects ordinal regression on global score versus content, layout, and participant specialty.

Predictor	β (SE)	z-score	*P* value	95% CI
Content^a^	.079 (0.008)	9.781	<.001	0.063 to 0.095
Layout^b^	.006 (0.011)	0.558	.58	−0.016 to 0.028
Specialization				
GP^c^	Reference	Reference	Reference	Reference
Specialist	−0.404 (0.380)	−1.062	.29	−1.149 to 0.341
Other caregiver	−0.544 (0.774)	−0.703	.48	−2.061 to 0.973

a Yes on the content question (%).

b Yes on the layout questions (%).

c GP=general practitioner.

### Consensus

Consensus for layout questions was high, with a median of 96.77% (IQR 90.22‐100.00). Content questions showed lower agreement (median 83.87%, IQR 67.74‐96.77). The overall consensus of both categories was 93.55% (IQR 80.65‐100.00; Appendix 7 in Multimedia Appendix 7). When examining the items in detail, consensus levels were substantially lower for the no categories (Appendix 7 in Multimedia Appendix 7, Figure 7.2 in Multimedia Appendix 7).

Category-level analysis showed that the *medication* section had the lowest consensus for both layout and content, while the *conclusion* section achieved perfect agreement for layout but lower for content (Table 4).

**Table 4.** Consensus by question category and type.

Question category	Content, median (IQR)	Layout, median (IQR)	All^a^ (content % layout), median (IQR)
General	82.3 (61.3‐96.8)	96.8 (83.9‐100)	96.8 (77.4‐100)
Medical history	91.9 (74.2‐100)	96.8 (90.3‐100)	96.8 (87.1‐100)
Medication	69.4 (58.1‐83.9)	93.5 (80.6‐100)	87.1 (71.0‐96.8)
Investigations	80.6 (65.3‐90.3)	100 (96.8‐100)	93.5 (74.2‐100)
Hospital course	88.7 (74.2‐93.5)	100 (93.5‐100)	96.8 (87.1‐100)
Follow-up	87.1 (71.0‐96.8)	96.8 (90.3‐100)	93.5 (87.1‐100)
Conclusion	82.3 (64.5‐93.5)	100 (96.8‐100)	98.4 (89.5‐100)

a Combination of content and layout questions.

Additional information can be found in Appendix 7 (Figure 7.3 and Figure 7.4) in Multimedia Appendix 7, which presents the consensus for each summary and question.

### Interrater Agreement

**Figure 5.**
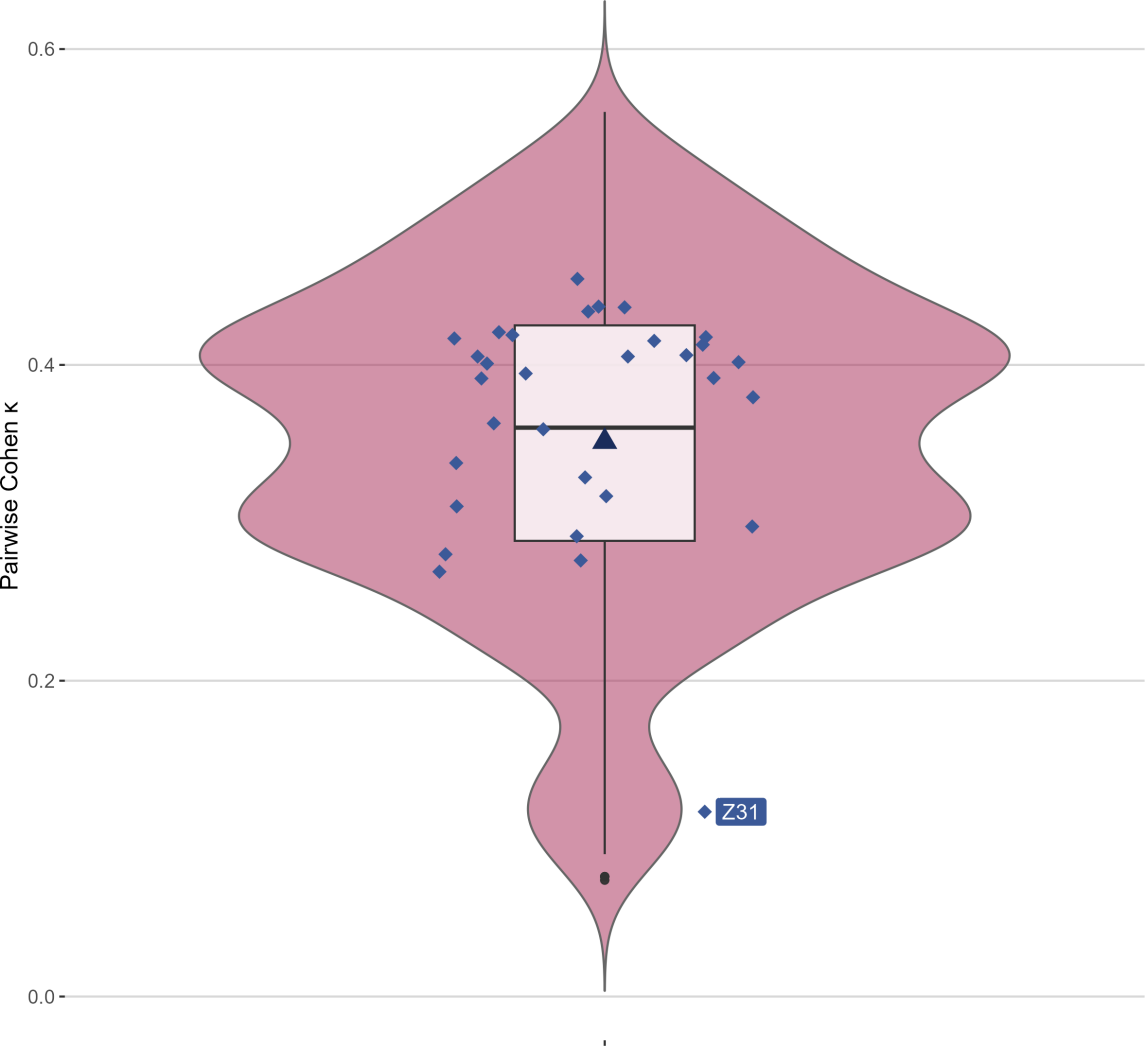
Pairwise Cohen κ between all participants. Diamonds represent median κ for each interrater; triangles represent mean pairwise Cohen κ. Z31=participant Z31.

Pairwise Cohen κ values ranged from 0.07 to 0.56, with a median of 0.36 (IQR: 0.29‐0.43), indicating generally low agreement among raters (Figure 5; Appendix 8.1 in Multimedia Appendix 8). Participant Z31 demonstrated notably lower agreement compared to others. A Dunn post hoc test confirmed significant differences for this participant (Appendix 8.2 in Multimedia Appendix 8).

The ICC for all questions combined was 0.945 (95% CI 0.942‐0.948). Layout questions achieved slightly higher ICC (0.961, 95% CI 0.958‐0.963), while content questions treated as binary yielded 0.908 (95% CI 0.899‐0.916). Excluding participant Z31 did not produce a significant difference in the ICC (Appendix 8.3 in Multimedia Appendix 8), so we continued including this participant in our analysis.

The ICC for global scores was 0.776 (95% CI 0.649‐0.875). Fleiss κ for content questions was lower (0.221, 95% CI 0.205‐0.237), with category-specific values ranging from 0.198 to 0.240 (Table 5).

**Table 5.** Interrater overview: intraclass correlation coefficient (ICC) for binary content and layout questions and Fleiss κ for categorical content questions.

Methods and group	Cohen κ	95 % CI
ICC		
Content (binary) and layout	0.945	0.942‐0.948
Layout	0.961	0.958‐0.963
Content—binary (yes/no)	0.908	0.899‐0.916
Fleiss		
Content—categorical	0.221	0.205‐0.237
OK	0.240	0.223‐0.257
Missing information	0.198	0.177‐0.218
Irrelevant details	0.202	0.170‐0.232
Hallucinations	0.235	0.173‐0.293

Further category-specific ICC analysis (Table 6; Appendix 8.4 in Multimedia Appendix 8) confirmed patterns observed in descriptive consensus measures. Interrater reliability was excellent across most sections, with median ICCs around 0.92 for the majority of items. The highest reliability was observed in the *medical history*, *follow-up*, and *conclusion* sections, where ICCs reached up to 0.96. The medication section showed comparatively lower, but still substantial, reliability for content (ICC=0.83). Notably, the *conclusion* section had a lower ICC for layout (0.65), indicating more variability among raters in assessing structural aspects of this section.

**Table 6.** Intraclass correlation coefficient layout and content question-by-question category.

Question category	Content	Layout	Content and layout
General	0.927 (0.902‐0.947)	0.904 (0.887‐0.920)	0.927 (0.916‐0.938)
Medical history	0.923 (0.910‐0.935)	0.977 (0.974‐0.980)	0.964 (0.960‐0.968)
Medication	0.825 (0.777‐0.867)	0.938 (0.926‐0.948)	0.919 (0.907‐0.930)
Investigations	0.870 (0.838‐0.898)	0.934 (0.918‐0.948)	0.919 (0.905‐0.931)
Hospital course	0.897 (0.868‐0.922)	0.915 (0.899‐0.929)	0.917 (0.904‐0.928)
Follow-up	0.900 (0.875‐0.922)	0.974 (0.970‐0.979)	0.955 (0.949‐0.961)
Conclusion	0.931 (0.904‐0.954)	0.654 (0.559‐0.737)	0.953 (0.942‐0.962)

Further inspection of the medication section showed that lower agreement among raters was partly associated with ambiguous prompt wording, particularly instructions such as “show temporary medication.” These ambiguities led evaluators to apply different interpretations of what information should be included in the summary, contributing to reduced consensus even in cases where the model output itself was not incorrect. This indicates that both the characteristics of the model output and the clarity of the prompt influenced the observed agreement patterns in this section.

### PI Analysis

The PI revealed substantial differences between layout and content items. Layout questions exhibited a high degree of prevalence skew, with an overall PI of 0.768 and a mean PI of 0.856 (SD 0.211). Several categories showed PI values above 0.90, indicating that layout-related items were almost always rated identically (typically “yes”), resulting in limited variability across raters. Content items demonstrated more balanced response distributions, with an overall PI of 0.550 and a mean PI of 0.622 (SD 0.316). Category-specific PI values ranged from 0.430 (medication) to 0.720 (medical history), reflecting greater heterogeneity in item difficulty and a broader range of rater judgments.

A detailed overview of PI values per category is presented in Table 7.

**Table 7.** Overview of prevalence index (PI) values for layout and content items by question category.

Category	Layout PI^a^, mean (SD)	Content PI^b^, mean (SD)
General	0.824 (0.244)	0.573 (0.351)
Conclusion	0.956 (0.087)	0.573 (0.294)
Follow-up	0.886 (0.160)	0.665 (0.274)
Medication	0.754 (0.258)	0.430 (0.282)
Investigations	0.905 (0.196)	0.545 (0.300)
Hospital course	0.875 (0.227)	0.675 (0.282)
Medical history	0.864 (0.178)	0.720 (0.317)

a Overall PI: 0.768; PI, mean (SD): 0.856 (0.211).

b Overall PI: 0.550; PI, mean (SD): 0.622 (0.316).

These findings confirm that layout items were highly homogeneous, while content items displayed more meaningful variability. Importantly, the high PI values for layout items help explain the discrepancy between the relatively low median Cohen κ and the high ICC: skewed response distributions can suppress κ, whereas ICC remains elevated because it is driven by between-item variance rather than categorical balance.

Finally, responses to the open-ended question were analyzed qualitatively and summarized by topic (Multimedia Appendix 9).

## Discussion

### Principal Findings

This study examined expert evaluation and interrater agreement in the assessment of LLM-generated discharge summaries, using the FRAIT framework, to assess structural fidelity, semantic accuracy, and usability.

Regarding the evaluation of structural integrity, layout adherence was consistently high, whereas content accuracy, particularly for medication details, remains a critical challenge. These findings align with prior research on clinical text summarization, which identifies medication and follow-up instructions as frequent sources of error and hallucination [9,10]. Although layout adherence was 88%, the remaining 12% reflects structural inconsistencies that may hinder automated processing and pose challenges for electronic health record integration. Even infrequent deviations from required section formatting can disrupt downstream workflow steps, indicating that layout performance should be considered a potential vulnerability rather than a strength.

The strong association between the positive response rate for content and global scores underscores the importance of semantic integrity in clinical documentation. Evaluators prioritized correctness over format, reinforcing FRAIT’s emphasis on clinically meaningful metrics rather than purely structural criteria. Future iterations should therefore incorporate automated validation of medication-related information. This also aligns with the context-aware evaluation by Agrawal et al [6].

Interrater reliability analysis revealed high ICC values for global scores but low pairwise agreement for individual questions, especially in complex categories such as medication. Our PI findings confirm that the discrepancy between item-level κ and overall ICC is partly attributable to prevalence skew in layout items, rather than true inconsistency in evaluator behavior. This suggests that while evaluators share similar global impressions, item-level judgments are subject to interpretation variability, a challenge also noted in previous discharge summary studies. A deeper analysis of the medication section revealed that ambiguous prompts (eg, “show temporary medication”) contributed to low agreement.

The medication section warrants particular attention, as it exhibited the highest hallucination rate (7.8%) among all content categories. A deeper review showed, however, that most of these hallucinations were related to start, stop, or change medication status rather than incorrect drug content. For example, in one case, vitamin D was listed on admission but not repeated in the discharge medication; the model labeled it as “unchanged,” which some raters considered a hallucination because no corresponding entry was present. This section also showed the lowest positive response rate and weakest interrater consensus, reflecting both its clinical complexity and the fact that some prompt instructions were interpreted differently by different raters. These findings highlight that LLM-generated medication summaries cannot be relied upon without human validation. When using this model and prompt configuration, human-in-the-loop verification remains essential to ensure clinical safety, and future implementations should include targeted safeguards, such as automated checks for omissions and medication-related inconsistencies. This highlights the need for clearer, context-specific prompts to optimize both LLM output and evaluation consistency, as suggested by Borse et al [11]. Follow-up workshops were organized to refine prompt design and ensure unambiguous interpretation, which is essential for improving interrater agreement.

The PI analysis clarifies the discrepancy between the moderate median Cohen κ (0.36) and the high ICC (0.945). Layout items showed consistently high PI values, indicating minimal variability and near-uniform “yes” responses. Such skewed distributions suppress κ (the prevalence paradox [12]) while inflating ICC, which is driven by between-item variance rather than categorical balance. Content items showed lower and more variable PI values, reflecting a more balanced mix of responses and greater item-level complexity. This explains why κ was lower for content judgments: raters disagreed more frequently on clinically nuanced items, although overall rating patterns remained consistent enough to keep ICC high. Together, these findings show that the high ICC partly reflects the high prevalence of affirmative responses in structural items rather than purely strong interrater consistency. Reporting PI alongside κ and ICC therefore provides a more accurate basis for interpreting agreement patterns.

Usability enhancements generated during the workshop, such as keyboard shortcuts, progress indicators, and autosave, reflect user-centered design principles and are expected to improve efficiency and reduce evaluator fatigue. These improvements support FRAIT’s goal of creating a scalable, reproducible evaluation environment for clinical LLMs.

An additional observation concerns infrastructure and security considerations. Although these aspects were outside the scope of this article, they represented the largest portion of the project’s budget. Establishing a secure, cloud-based environment was essential to ensure compliance with data protection standards and enable safe collaboration. This underscores the resource-intensive nature of deploying LLMs in clinical settings and highlights the importance of planning for robust infrastructure early in implementation.

While our findings confirm earlier reports of promising outcomes for LLMs in clinical documentation [9,10,13], infrastructure and human factors remain critical determinants of success. Addressing these challenges will be essential for scaling LLM-based solutions in health care.

### Limitations

A key limitation of this study is the exclusive use of a single standardized prompt and a single LLM (GPT-4o). Although GPT-4o is recognized for its advanced capabilities, our findings should not be generalized to other model families, including open-weight systems or medical-specific models, nor to alternative prompting strategies. While this controlled design was necessary to ensure consistent comparison across evaluators, it also restricts the generalizability of our findings. LLM performance evolves rapidly, and outputs can vary substantially across models, model versions, and prompt formulations. As a result, this study should be interpreted primarily as a validation of the FRAIT evaluation framework, rather than a definitive benchmark of LLM summarization performance.

As our task is clinical summarization, it is plausible that smaller, domain-specific models trained on medical corpora could outperform general-purpose models on factuality, medication handling, and terminology precision while also reducing hallucination risk and computational overhead. The growing adoption of domain-specific models, such as Meditron and HuatuoGPT, which have demonstrated strong performance in clinical contexts [1], suggests that specialized models may offer advantages in higher positive response rates and interpretability that general-purpose models cannot match. Furthermore, larger general-purpose LLMs may introduce unnecessary complexity and increase the risk of hallucinations compared to smaller, clinically optimized models, as noted by Lehman et al [14]. These considerations highlight the need for comparative studies involving multiple LLMs to better understand the generalizability, safety, and optimal balance between model size and specialization in health care applications. We did not test such models here. Therefore, our results may underestimate the attainable accuracy and safety for targeted deployments.

Our evaluation used expert-curated synthetic discharge letters, developed to maintain clinical plausibility while omitting direct identifiers. The original section structure and general layout were largely maintained; however, the transformation process may have reduced certain real-world irregularities such as incomplete phrasing, extraneous noise, or ambiguous references that commonly occur in routine documentation. This distribution shift may have resulted in inflated performance metrics, particularly regarding layout adherence, compared to those expected from more variable and unstructured real-world notes, and may similarly overstate content accuracy. Therefore, our results should be regarded as an upper bound established under controlled conditions, rather than indicative of field performance.

The free-text explanations accompanying “no” responses could not be systematically analyzed because many raters did not follow the intended instructions for marking errors. As a result, the raw annotations were inconsistent in format and therefore unsuitable for reliable qualitative or quantitative reporting. Although the rater pool included clinicians from different specialties, it may not fully capture the diversity of clinical perspectives. Variability in interpretation between GPs and hospital physicians, especially for nuanced categories such as medication details, could have influenced agreement scores and overall evaluations. Ensuring broader representation across specialties and experience levels would strengthen the reliability of future assessments.

Certain sections of the discharge summaries, such as the conclusion, contained limited data. This imbalance restricted the robustness of evaluation for those components and may have skewed the overall usability and accuracy metrics. Future research should aim for more balanced datasets to enable thorough analysis across all content categories. Furthermore, as multiple layout categories exhibited PI values exceeding 0.90, certain reliability measures, especially κ, should be interpreted with caution because high prevalence can restrict their capacity to distinguish genuine agreement from systematic response patterns.

Analysis of pairwise Cohen κ identified one rater whose agreement consistently differed from that of the other evaluators. A post hoc review indicated that delays in initiating and completing the evaluation tasks may have contributed to this variability. This finding highlights the importance of voluntary participation and adequate protected time for expert evaluation to support data quality integrity in future studies. Notably, overall interrater reliability remained high despite this variability, suggesting that the evaluation approach was robust under routine clinical conditions.

A reliable measure of time-on-task could not be obtained because participants were free to pause or take breaks while completing evaluations. Although the platform recorded start and end times, these time stamps do not reflect actual working time and therefore cannot be used to assess efficiency. This limits our ability to draw conclusions about the workflow impact of the evaluation process.

### Implications and Future Work

The findings of this study have several practical implications for the integration of LLMs into clinical workflows. While LLMs show promise in assisting with documentation tasks, they should not replace expert review. Human oversight remains essential to ensure patient safety and compliance with clinical standards. Automated quality assurance mechanisms, particularly those targeting omissions and hallucinations in critical areas, such as medication and follow-up instructions, will be vital for real-world deployment.

Prompt design emerged as a key factor influencing evaluation consistency. Ambiguous or poorly defined prompts contributed to variability in interrater agreement and model output quality. Future research should therefore prioritize the development of clear, context-specific prompts and explore adaptive prompt optimization strategies to enhance reliability.

Model refinement is another important area for future work. Comparative evaluations of multiple LLMs, including domain-specific models, are needed to identify configurations that balance positive response rate, interpretability, and computational efficiency. Additionally, longitudinal studies should assess the real-world impact of LLM-assisted documentation on clinical workflows, patient outcomes, and resource allocation. These investigations will provide critical insights into the scalability and sustainability of LLM integration in health care.

To ensure standardization, the evaluation was restricted to a single medical prompt, despite participants having previously developed personalized prompts in a separate workshop [2]. As emphasized by Verma et al [8], the value of summarization is maximized when outputs are tailored to the specific needs of different clinical roles and contexts. Customized summaries, adapted for specialists, general practitioners, or administrative staff, ensure that each user receives the most relevant and actionable information for their responsibilities. This approach not only improves usability but also supports safer and more effective integration of AI tools into diverse health care environments.

Building on recent insights from Croxford et al [7], future research should explore the potential of LLMs not only as tools for generating clinical documentation but also as evaluators, or “judges,” of their own outputs. They propose that context-aware LLMs, when properly calibrated and validated, could assist in the assessment of clinical summaries by providing rapid, scalable, and consistent feedback on accuracy, completeness, and relevance. Integrating LLMs as judges within expert-driven frameworks such as FRAIT may enhance the efficiency of evaluation processes, support continuous quality improvement, and facilitate benchmarking across diverse clinical scenarios. However, this approach will require rigorous validation to ensure that automated judgments align with clinical standards and expert consensus and that potential biases or limitations are systematically addressed.

### Conclusions

The FRAIT framework demonstrates strong potential for evaluating LLM-generated discharge summaries, offering a structured approach that balances usability and clinical relevance. While layout fidelity was consistently high, content accuracy, particularly for medication details, remains a critical challenge. Interrater reliability findings highlight the need for clearer prompts and standardized evaluation criteria to reduce variability. Usability enhancements developed through co-creation with clinicians further support FRAIT’s scalability and practical adoption. Overall, LLMs show promise as an aid in clinical documentation, but robust validation mechanisms and expert oversight are essential to ensure safety and accuracy. Future work should focus on prompt optimization, model refinement, and automated quality checks to advance reliable integration of LLMs into health care workflows.

## Supplementary material

10.2196/90374 Multimedia Appendix 1 Quest flow.

10.2196/90374 Multimedia Appendix 2 Evaluation questions by category.

10.2196/90374 Multimedia Appendix 3 Protocol synthetic discharge summaries FRAIT.

10.2196/90374 Multimedia Appendix 4 Sensitivity analysis.

10.2196/90374 Multimedia Appendix 5 Evaluation tool—extra feature list.

10.2196/90374 Multimedia Appendix 6 Overview global score.

10.2196/90374 Multimedia Appendix 7 Consensus.

10.2196/90374 Multimedia Appendix 8 Interrater agreement.

10.2196/90374 Multimedia Appendix 9 Overview answer open question.

## Abbreviations

DPO: Data Protection Officer
FRAIT: Framework and Implementation of AI Tools
ICC: intraclass correlation coefficient
LLM: large language model
PI: prevalence index
QUEST: Quality of Information, Understanding and Reasoning, Expression Style and Persona, Safety and Harm, and Trust and Confidence
