# Supplementary material for: Expert Evaluation and Consensus on GPT-4o Summaries of Clinical Letters: Validation and Results of the Framework and Implementation of AI Tools Project
Source: JMIR Med Inform. 2026 May 11;14:e90374. doi: 10.2196/90374 (PMC13160486; doi:10.2196/90374)
Supplement: Multimedia Appendix 1 [file medinform-v14-e90374-s001.pdf]

Appendix 4 – Evaluation tool – extra feature list

Table 4.1 Feature list

Category	Features
**Authentication & Authorization**	- Separate Auth0 tenant<br>- Azure AD sync with Auth0<br>- Manage permissions via DocAT<br>- Automatic role assignment using Azure AD groups (disabled)<br>- Auto llm-user permissions for valid users
**Infrastructure**	- Azure-OpenAI model container<br>- Pre-annotate container<br>- Kubernetes manager to spawn/remove worker pods
**Application Features**	- Meta-annotations: users can annotate model annotations (e.g., summaries)<br>• QA – evaluation<br>• Question-based<br>• Binary questions<br>• Open questions<br>• Binary open questions<br>• Questions with text highlights<br>• Multiple choice questions (Category, Subject)<br>- Auto save
**Visual Features**	- QA – evaluation view<br>• Progress bar<br>• Auto scroll<br>• Helper functions<br>• Full screen for source text and summary<br>• Shortcuts<br>• Text highlighting<br>- Markdown rendering<br>- Shielded Swagger
**Miscellaneous**	- Contact DSI button<br>- Custom login screen<br>- Light DocAT client library for interfacing with API

Legend: AD=Active Directory; QA=Question & Answer; DSI=Data Science Institute

Figure 4.2 Evaluation tool - screenshots

Evaluation Tool – without ovelays

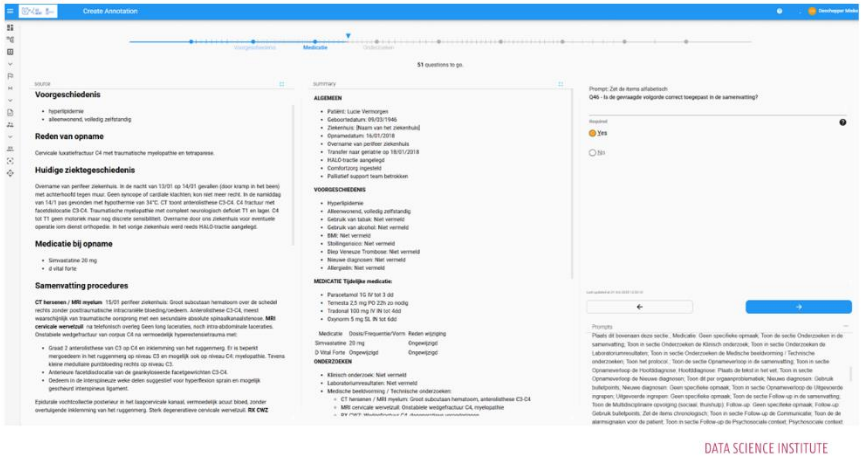

Evaluation Tool - with overlays

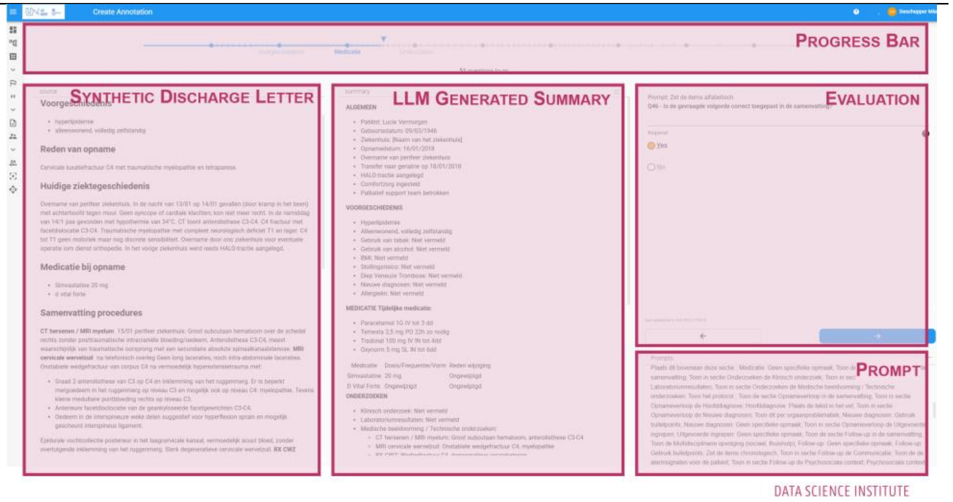

Figure 4.3 Evaluation tool screenshots - Example Content – answer No

FIGURE 4.3.1 MISSING INFORMATION

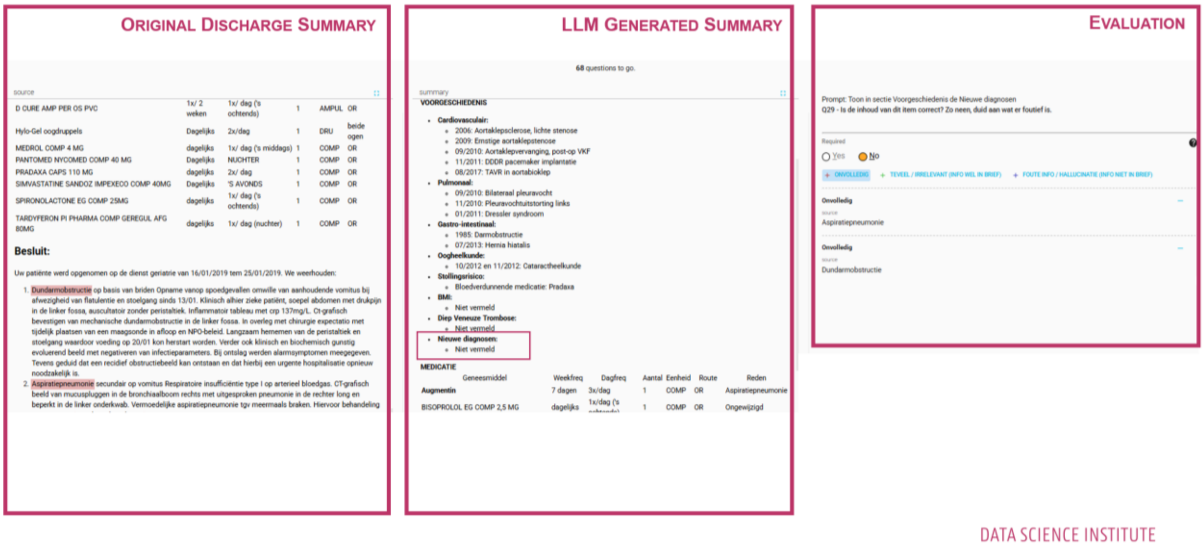

FIGURE 4.3.2 IRRELEVANT DETAILS

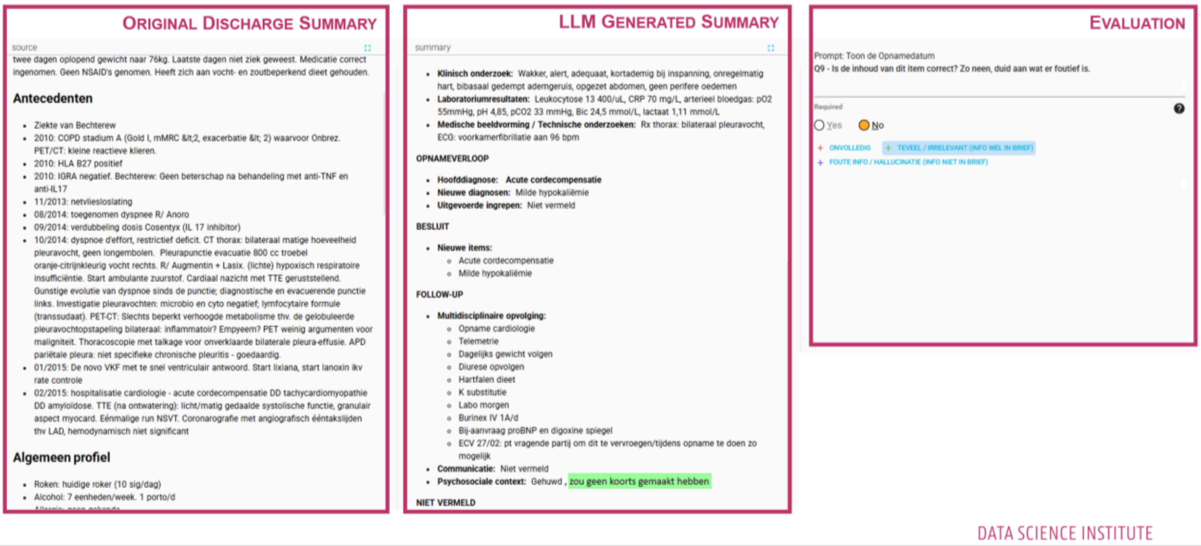

FIGURE 4.3.3 HALLUCINATION

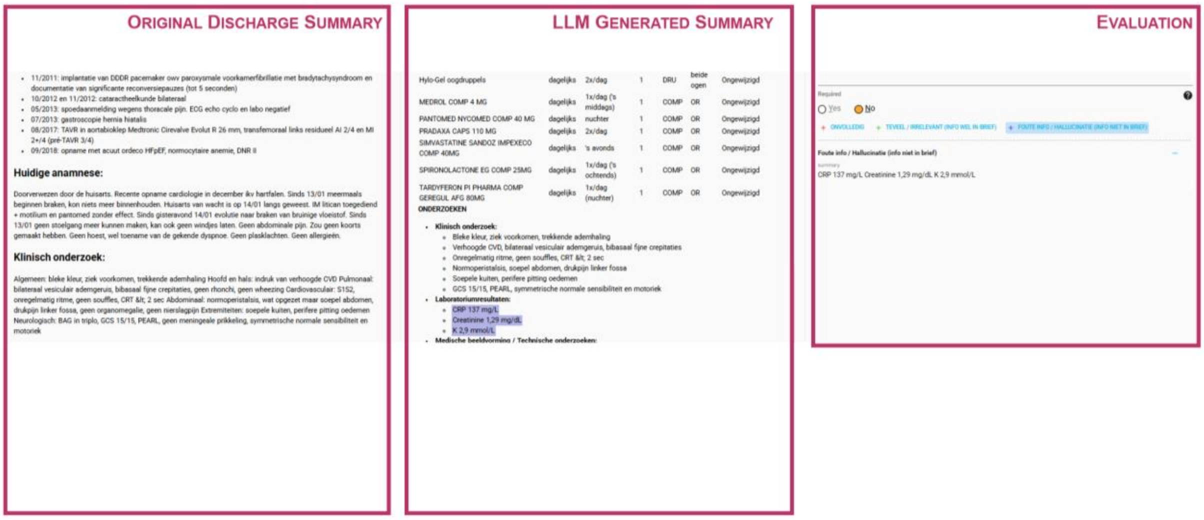

DATA SCIENCE INSTITUTE
