# Supplementary material for: Expert Evaluation and Consensus on GPT-4o Summaries of Clinical Letters: Validation and Results of the Framework and Implementation of AI Tools Project
Source: JMIR Med Inform. 2026 May 11;14:e90374. doi: 10.2196/90374 (PMC13160486; doi:10.2196/90374)
Supplement: Multimedia Appendix 2 [file medinform-v14-e90374-s002.pdf]

Appendix 2 - Evaluation questions by category

Table 2.1 Evaluation questions by category

Type	Category	N	Percentage
**Content**	General	3	3.2
	Medical History	10	10.6
	Medication	4	4.3
	Investigations	5	5.3
	Hospital course	4	4.3
	Follow-up	5	5.3
	Conclusion	2	2.1
**Layout**	General	9	9.6
	Medical History	16	17.0
	Medication	9	9.6
	Investigations	5	5.3
	Hospital course	9	9.6
	Follow-up	9	9.6
	Conclusion	4	4.3

Table 2.2 Overview questions

In below all different questions are listed.

Layout:

- Has the requested formatting been applied?
- Is the requested order correctly applied in the summary?
- Is the formatting as requested?
- Is the summary written in the requested language?
- Is the total length of the summary as requested?
- Is the requested section included in the summary?
- Is the requested item included in the summary?
- Are the dates in the correct format?
- Are the requested items present?

Content:

- Is the content of this item correct? If not, specify what is incorrect.
- Have you been able to indicate all substantive errors in this section? If not, additionally specify what is still incorrect.

UZ GENT
UNIVERSITEIT GENT
Universitair Ziekenhuis Gent
C. Heymanslaan 10 | B 9000 Gent
www.uzgent.be

Examples:

**Prompt**	**Evaluation Question Layout**	**Evaluation Question Content**
Show the changed medication (dose, frequency, shape)	Is the requested item included in the summary?	Is the content of this item correct? If not, specify what is incorrect.
Medication: Place the text in bold	Is the formatting as requested?	
