# Supplementary material for: Expert Evaluation and Consensus on GPT-4o Summaries of Clinical Letters: Validation and Results of the Framework and Implementation of AI Tools Project
Source: JMIR Med Inform. 2026 May 11;14:e90374. doi: 10.2196/90374 (PMC13160486; doi:10.2196/90374)
Supplement: Multimedia Appendix 3 [file medinform-v14-e90374-s003.pdf]

Appendix 3 – Protocol Synthetic Discharge Summaries FRAIT

(Original protocol in Dutch, translated to English using GenAI).

Objective
Develop discharge letters that do not contain identifiable patient information for the FRAIT project, while still including relevant medical details. These letters will be made available to FRAIT participants, in compliance with GDPR regulations. Participants will use these letters to evaluate the developed tool.

Step-by-Step Plan
Step 1: Approval by Data Access Committee / Medical Ethics Committee
Obtain approval from the Data Access Committee and the Medical Ethics Committee (CME) to work with synthesized letters created by the DSI for the FRAIT project.
Justification for this research is based on legitimate interest as the legal basis.
This assessment was carried out in three steps:

- **Purpose Test:** Gain scientific insights and improve care quality (by providing necessary data to healthcare providers more efficiently).
- **Necessity Test:** Realistic letters are essential to achieve the intended goal and conduct a thorough evaluation. A public pool of discharge letters is available for this research.
- **Balancing of Interests:** Using a step-by-step approach, the recognizability of letters is minimized. Selection starts from an existing care relationship. Letters do not contain highly sensitive topics such as psychiatry or gender issues. All patients have been deceased for a considerable time.

Approach for creating synthesized letters:
The patient's discharge letter is anonymized through multiple steps, followed by extensive content adjustments. This includes removing direct identifiers and modifying medically irrelevant details for tool evaluation. Key clinical data necessary for tool evaluation are retained or altered so that no identifiable clinical information remains.

Step 2: Patient Selection Criteria

- Age ≥ 18 years
- Deceased
- Care relationship with the patient by the physician who identified the patients

Step 3: Selection of Discharge Letters

- Database: Gusta (contains no letters from cardiology or maternity)
- Filter only discharge letters (DOCUMENTTYPE = 'ONT')
- Exclude psychiatry department letters (DIENST != 'PSYCH')
- Only completed letters (status = 2)
- Only verified letters (VERIFIED = 200)
- Exclude letters specifically about death ('SJABLOON' does not contain 'overlijden')

Step 4: Random sample of 500 letters

Step 5: Synthesizing the Letters

1. Convert text file to Word document
2. Remove headers and footers
   - Delete everything above the salutation (“Dear…”) and below “Sincerely” or “With collegial regards,” including hospital and patient identifiers.
3. Replace patient’s first and last name
   - Replace with random names while preserving gender (important for pathology).
     Example: Luc Vermorgen → Bernard De Vuyst
4. Remove address details
5. Replace date of birth
   - Replace day and month randomly; adjust year by a few years to keep age realistic for pathology.
     Example: 05/06/1956 → 02/12/1958
6. Make medical content less recognizable
   - Replace, remove, or add diagnoses without affecting relevant content.
     Example: “left knee prosthesis” → “right hip prosthesis.”
     Rare conditions (e.g., Gaucher disease) removed if irrelevant.
7. Handle date references
   - **Data shifting:** Adjust all years by up to 10 years while keeping relative timing intact.
   - Remove precision in medical history: keep only year or month/year formats (apply data shifting).
     Example: 15/05/2017 → 05/2017
   - For current episode, keep day/month if relevant.
8. Remove or replace sensitive psychological/social data
9. Remove names of doctors and hospitals

Alternative Route for Providing Synthesized Letters
Participating healthcare providers may optionally create synthetic letters based on discharge letters of their living patients, provided they deliver an information sheet to the patient. This protocol is available on the FRAIT information channel.

Attachment: Complete list of PII data (as defined by Ethics Committee UZ Gent) and approach for FRAIT.
PII | Actions

- **Country/State** → remove
- **Race** → remove
- **Postal code, city** → remove
- **Job situation and exposure to risk factors** → remove, unless relevant for the diagnosis
- **Gender** → keep, as it is relevant for diagnostics
- **Age** → remove
- **Login credentials** → remove

- **First and last name** → replace with a random name, depending on gender
- **Date of birth** → replace with another date of birth, differing by several years
- **Dates** → adjust via data shifting: shift years by max. 10 years
- **Initials of first and last name** → remove, possibly replace with “your patient(s)”
- **Home address** → remove
- **Email address** → remove
- **Phone numbers** → remove
- **Credit card numbers** → remove
- **Patient identification numbers** → remove
- **Driver’s license numbers** → remove
- **Identity card numbers** → remove
- **National registry number** → remove
- **Genetic data** → remove
- **Numbers (e.g., lab orders)** → remove
- **Non-personal time indications** → remove
