# Supplementary material for: Expert Evaluation and Consensus on GPT-4o Summaries of Clinical Letters: Validation and Results of the Framework and Implementation of AI Tools Project
Source: JMIR Med Inform. 2026 May 11;14:e90374. doi: 10.2196/90374 (PMC13160486; doi:10.2196/90374)
Supplement: Multimedia Appendix 4 [file medinform-v14-e90374-s004.pdf]

Appendix 4 Sensitivity Analysis: Comparison between the Full Rater Group and the Physician-only Subset.

Method

To ensure the robustness of the findings and to assess whether the professional background of the raters influenced the outcomes, a sensitivity analysis was performed. We compared the primary outcomes of the full rater cohort (N=31, including two non-physician experts) against a subset consisting exclusively of physicians (N=29).

Results

The sensitivity analysis demonstrated that the inclusion of non-physician raters had a negligible impact on both the descriptive scores and the reliability metrics (Table IX.1). The Intraclass Correlation Coefficient (ICC) for the full group was 0.945 (95% CI [0.942, 0.948]), which remained virtually unchanged when restricted to physicians (ICC = 0.944, 95\% CI [0.941, 0.947]). Similarly, overall PI showed only minor fluctuations, with the largest difference observed in the Content category (a decrease of 0.012). The high degree of overlap between the 95% confidence intervals across all metrics indicates that the results are highly robust and not driven by the specific background of the two non-physician raters.

**Category**	**Metric**	**Full Group (N=31)**	**Physician Subset (N=29)**
**Scores (%)**	Overall Score	68.90 [68.85, 68.94]	68.71 [68.66, 68.75]
**% OK**	Content	77.50	76.91
	Layout	88.39	88.48
**ICC**	Layout + Content	0.945 [0.942, 0.948]	0.944 [0.941, 0.947]
	Layout	0.961 [0.958, 0.963]	0.959 [0.956, 0.962]
	Content	0.901 [0.899, 0.916]	0.908 [0.890, 0.917]
**PI overall**	Layout + Content	0.691	0.688
	Layout	0.768	0.770
	Content	0.550	0.538

Table 4.1 Sensitivity analysis
Legend: ICC = Intraclass Correlation Coefficient; CI = 95% Confidence Interval; PI = Prevalence Index.
