# Supplementary material for: Expert Evaluation and Consensus on GPT-4o Summaries of Clinical Letters: Validation and Results of the Framework and Implementation of AI Tools Project
Source: JMIR Med Inform. 2026 May 11;14:e90374. doi: 10.2196/90374 (PMC13160486; doi:10.2196/90374)
Supplement: Multimedia Appendix 5 [file medinform-v14-e90374-s005.pdf]

# Appendix 5 – Evaluation tool – extra feature list

### Table 5.1 Feature list

| Category | Features |
|---|---|
| **Authentication & Authorization** | - Separate Auth0 tenant<br>- Azure AD sync with Auth0<br>- Manage permissions via DocAT<br>- Automatic role assignment using Azure AD groups (disabled)<br>- Auto llm-user permissions for valid users |
| **Infrastructure** | - Azure-OpenAI model container<br>- Pre-annotate container<br>- Kubernetes manager to spawn/remove worker pods |
| **Application Features** | - Meta-annotations: users can annotate model annotations (e.g., summaries)<br>• QA – evaluation<br>• Question-based<br>• Binary questions<br>• Open questions<br>• Binary open questions<br>• Questions with text highlights<br>• Multiple choice questions (Category, Subject)<br>- Auto save |
| **Visual Features** | - QA – evaluation view<br>• Progress bar<br>• Auto scroll<br>• Helper functions<br>• Full screen for source text and summary<br>• Shortcuts<br>• Text highlighting<br>- Markdown rendering<br>- Shielded Swagger |
| **Miscellaneous** | - Contact DSI button<br>- Custom login screen<br>- Light DocAT client library for interfacing with API |

Legend: AD=Active Directory; QA=Question & Answer; DSI=Data Science Institute

# Figure 5.2 Evaluation tool - screenshots

**Evaluation Tool – without ovelays**

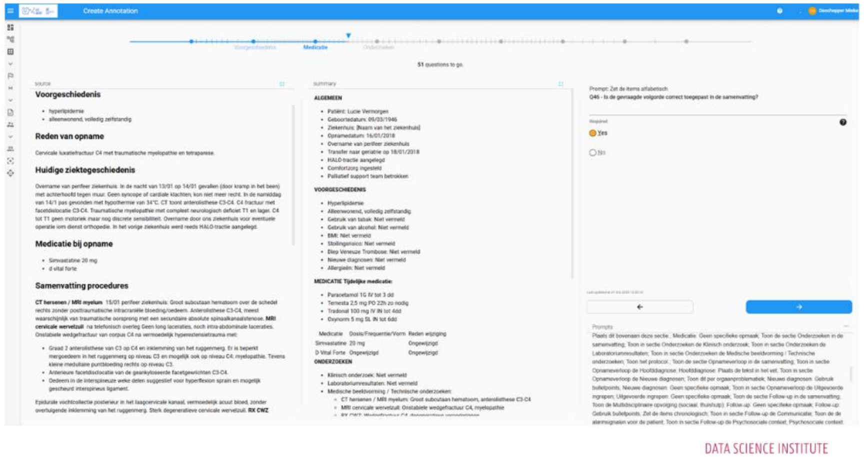


**Evaluation Tool - with overlays**

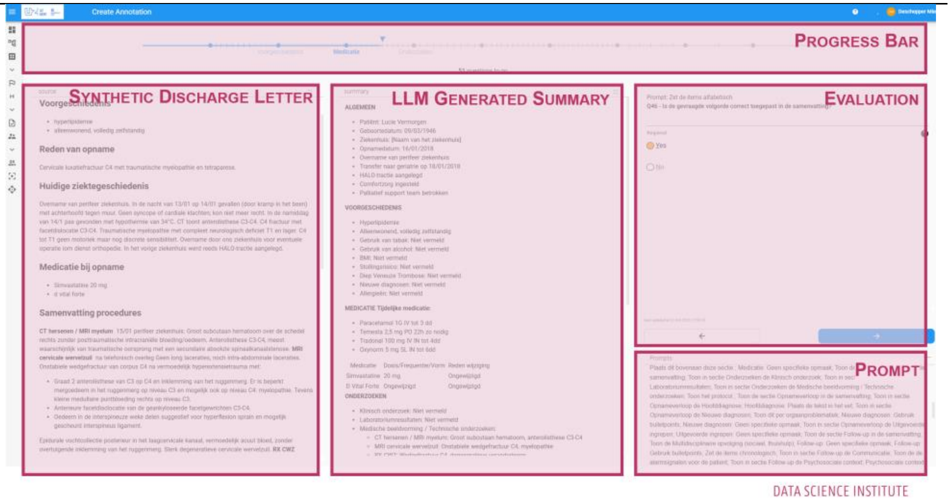

# Figure 5.3 Evaluation tool screenshots - Example Content – answer No

## FIGURE 5.3.1 MISSING INFORMATION

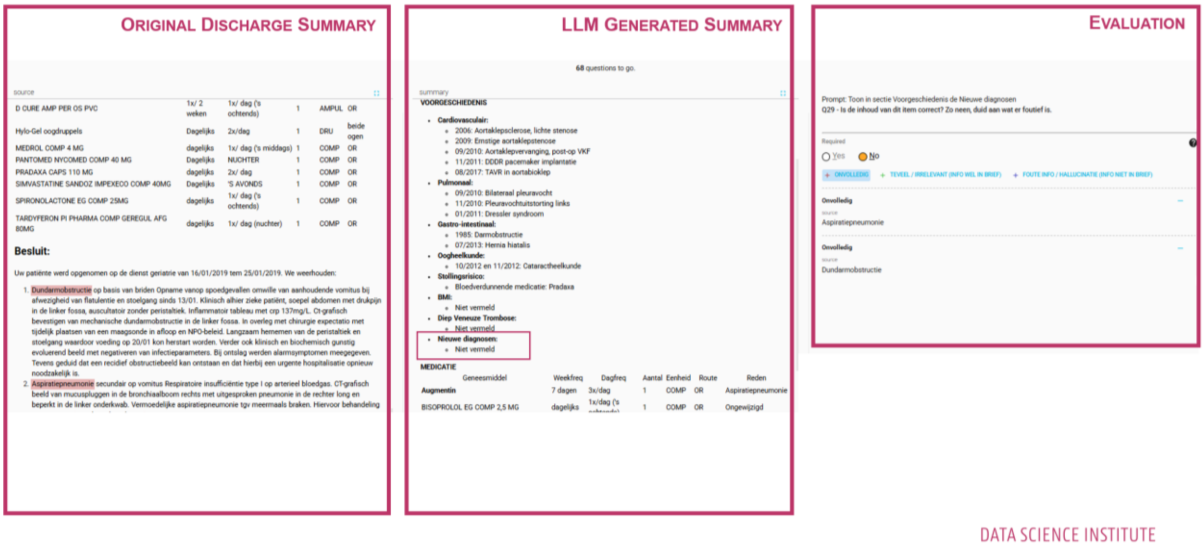


## FIGURE 5.3.2 IRRELEVANT DETAILS

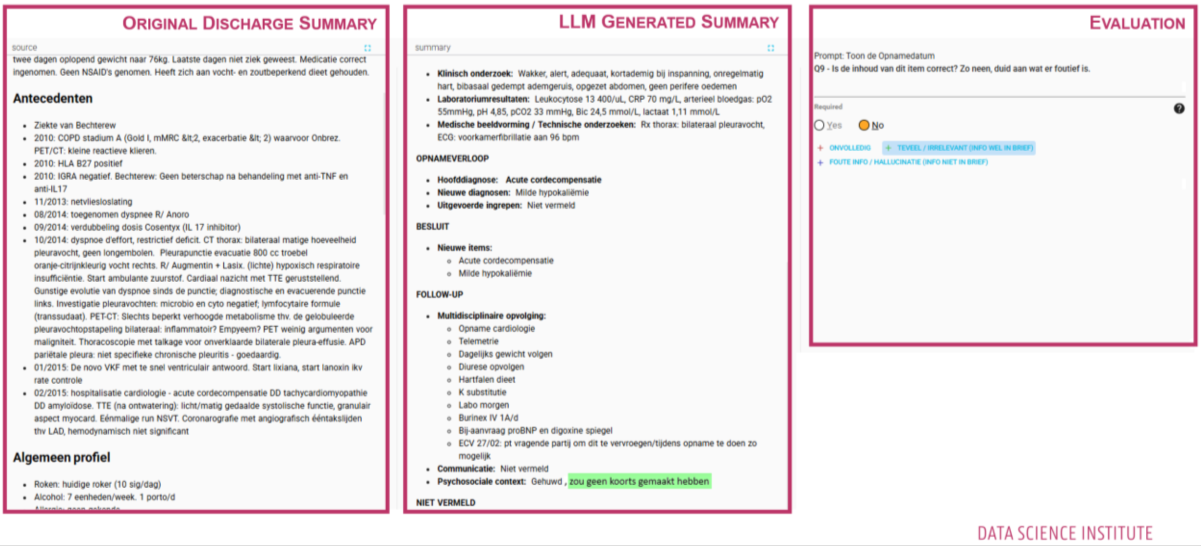


UZ GENT
UNIVERSITEIT GENT
Universitair Ziekenhuis Gent
C. Heymanslaan 10 | B 9000 Gent
**www.uzgent.be**

### FIGURE 5.3.3 HALLUCINATION

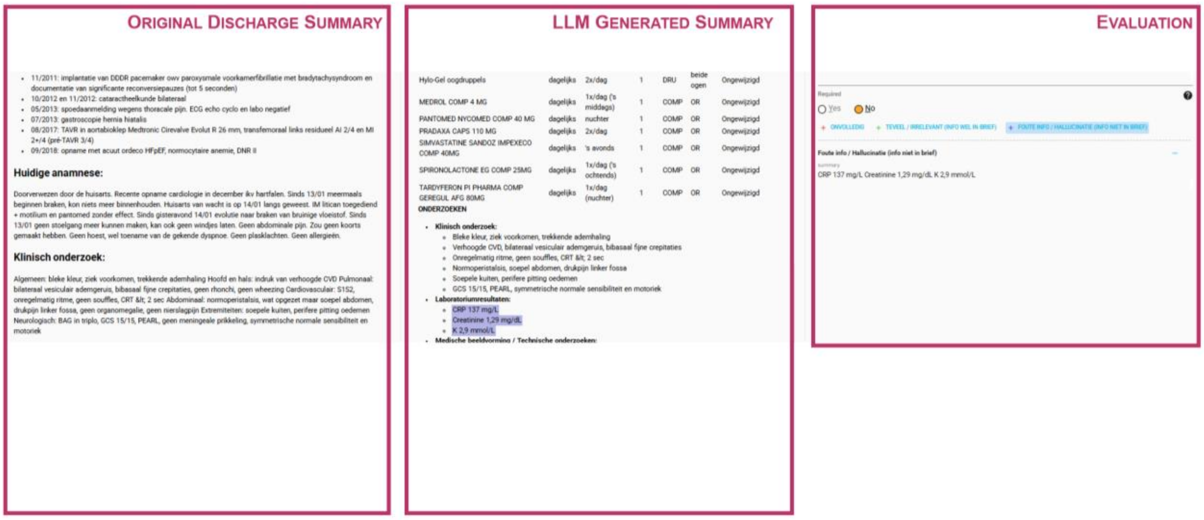


DATA SCIENCE INSTITUTE

UZ GENT
UNIVERSITEIT GENT
Universitair Ziekenhuis Gent
C. Heymanslaan 10 | B 9000 Gent
**www.uzgent.be**
