# Supplementary figures and images for: Expert Evaluation and Consensus on GPT-4o Summaries of Clinical Letters: Validation and Results of the Framework and Implementation of AI Tools Project
Source: JMIR Med Inform. 2026 May 11;14:e90374. doi: 10.2196/90374 (PMC13160486; doi:10.2196/90374)

## Appendix 6 – Overview Global Score

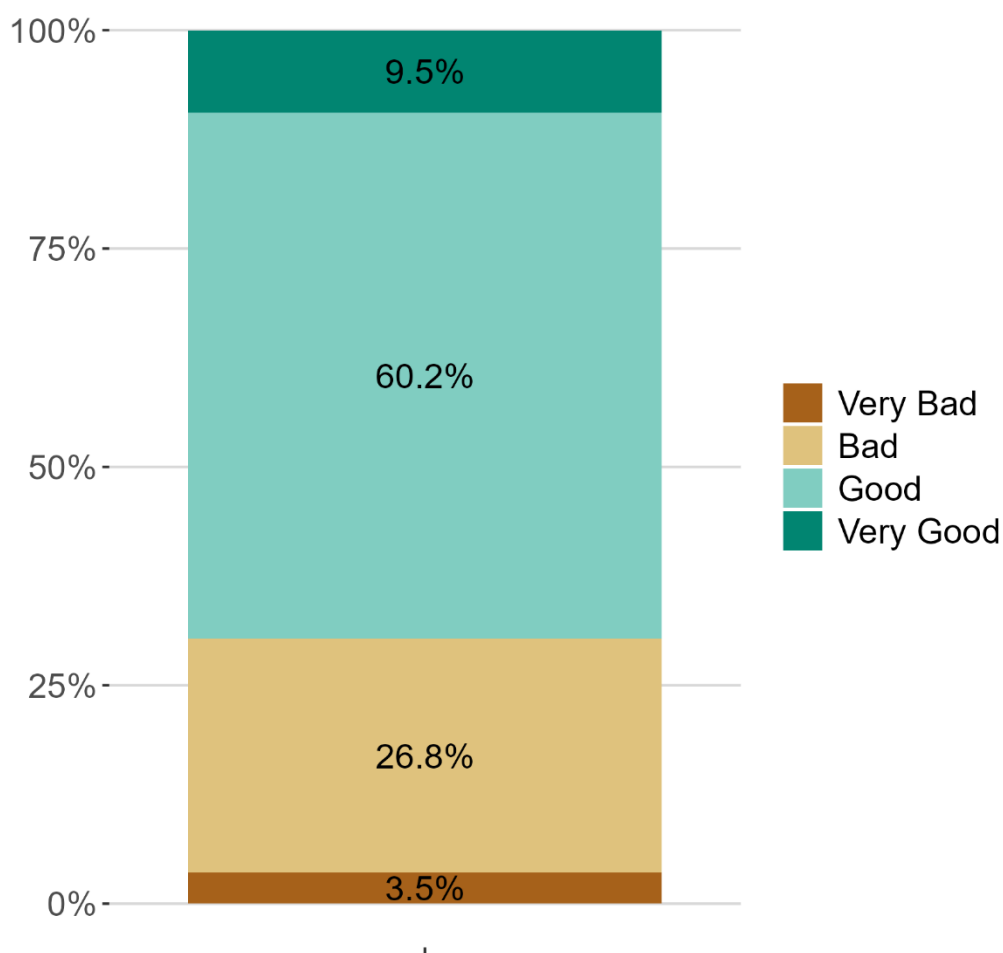

Supplement: Multimedia Appendix 6 [file medinform-v14-e90374-s006.pdf]
