# Supplementary material for: Expert Evaluation and Consensus on GPT-4o Summaries of Clinical Letters: Validation and Results of the Framework and Implementation of AI Tools Project
Source: JMIR Med Inform. 2026 May 11;14:e90374. doi: 10.2196/90374 (PMC13160486; doi:10.2196/90374)

# Appendix 7 – Consensus

In the figures below (Figure 7.1 & Figure 7.2) we show for each summary (y-axis) for each question (x-axis) the percentage consensus. For each summary also the total percentage consensus is shown.

## Figure 7.1 % Consensus for each question type

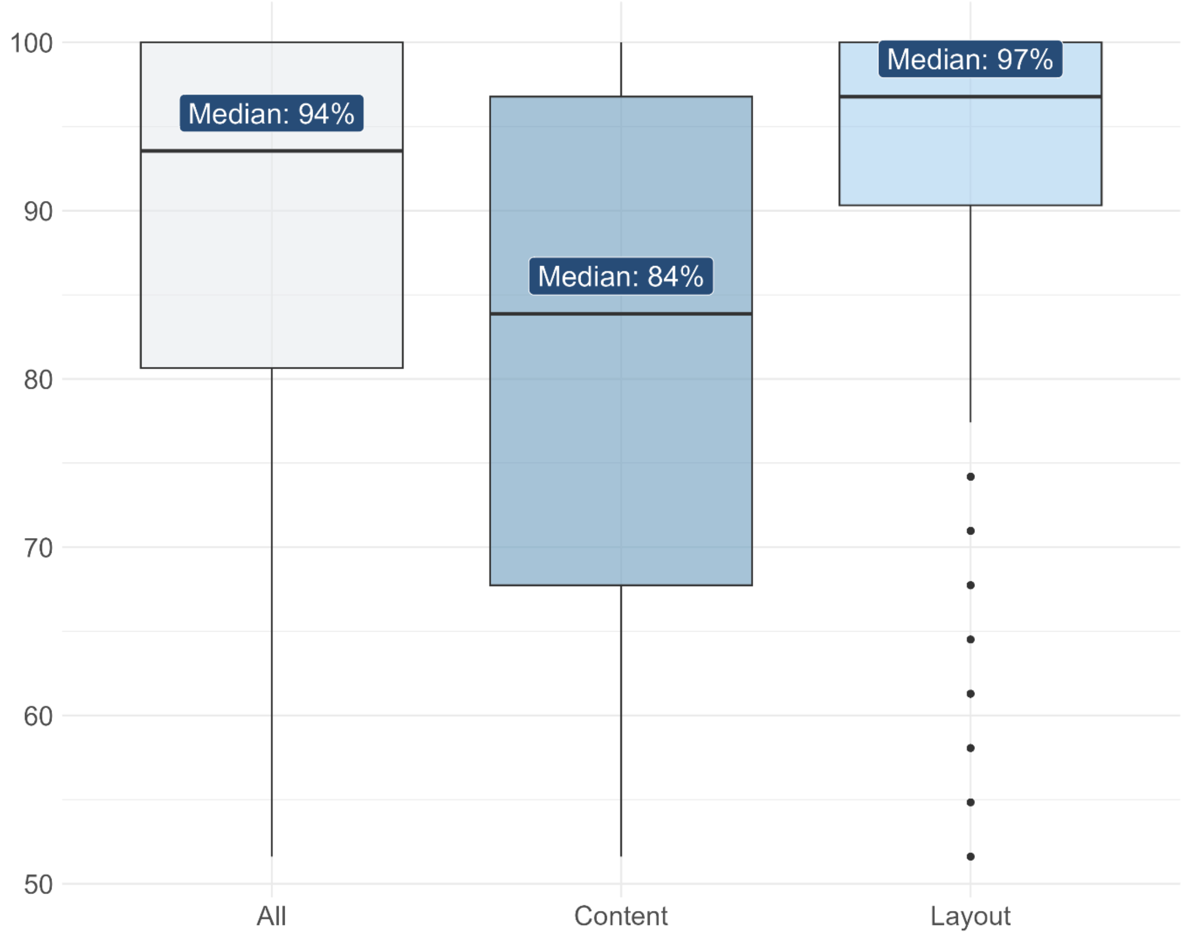

## Figure 7.2 % Median consensus for each answer type

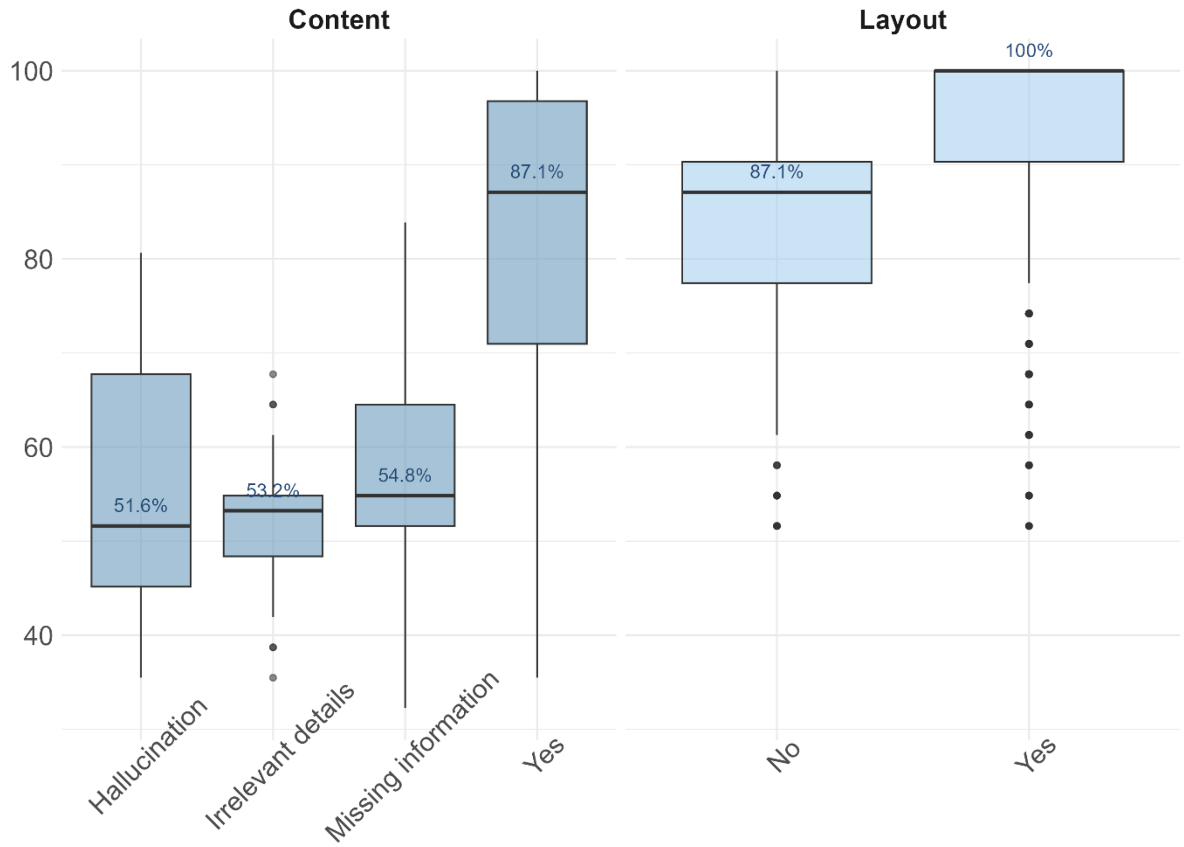

## Figure 7.3 Percentage Consensus for each summary and each Content question

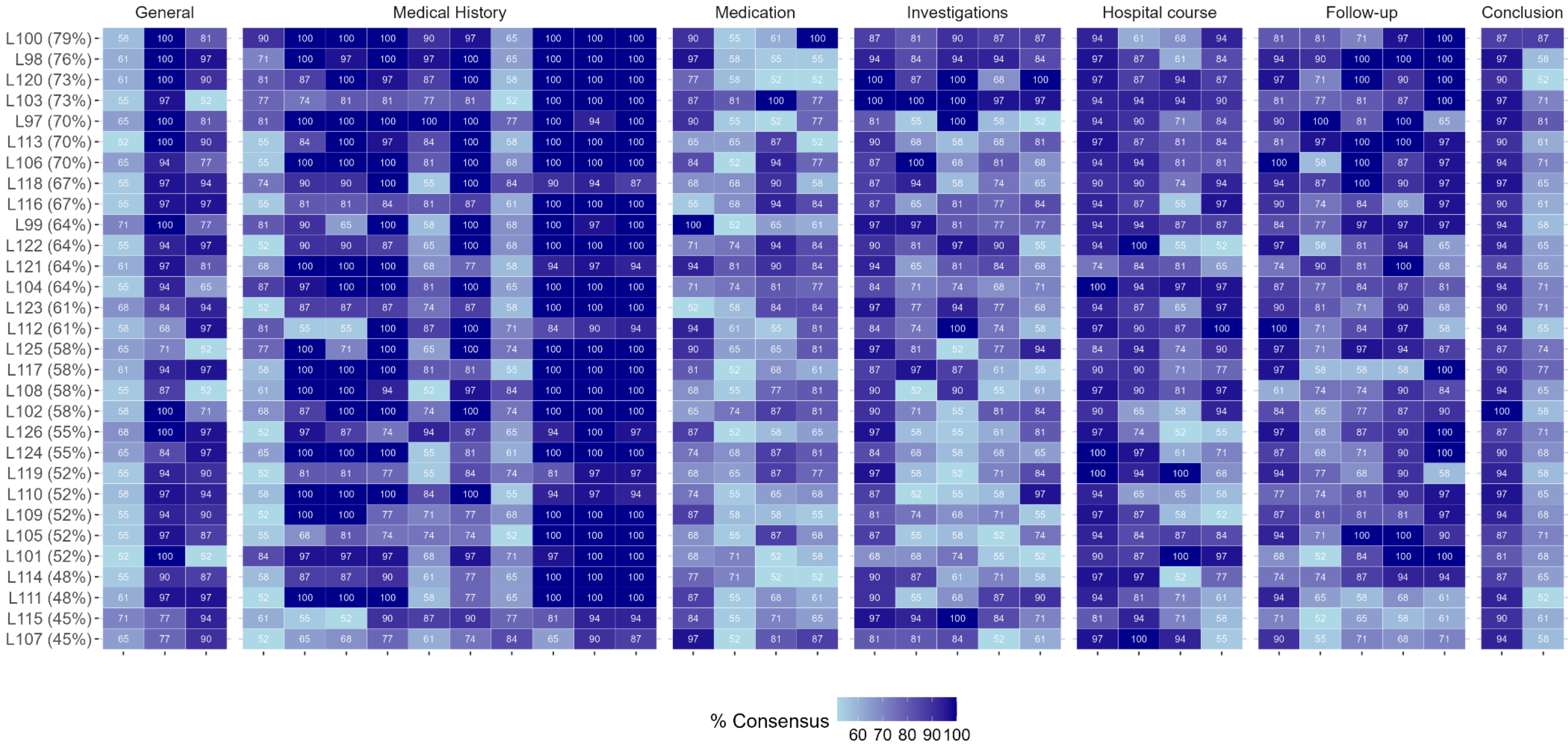

## Figure 7.4 Percentage Consensus for each summary and each Layout question

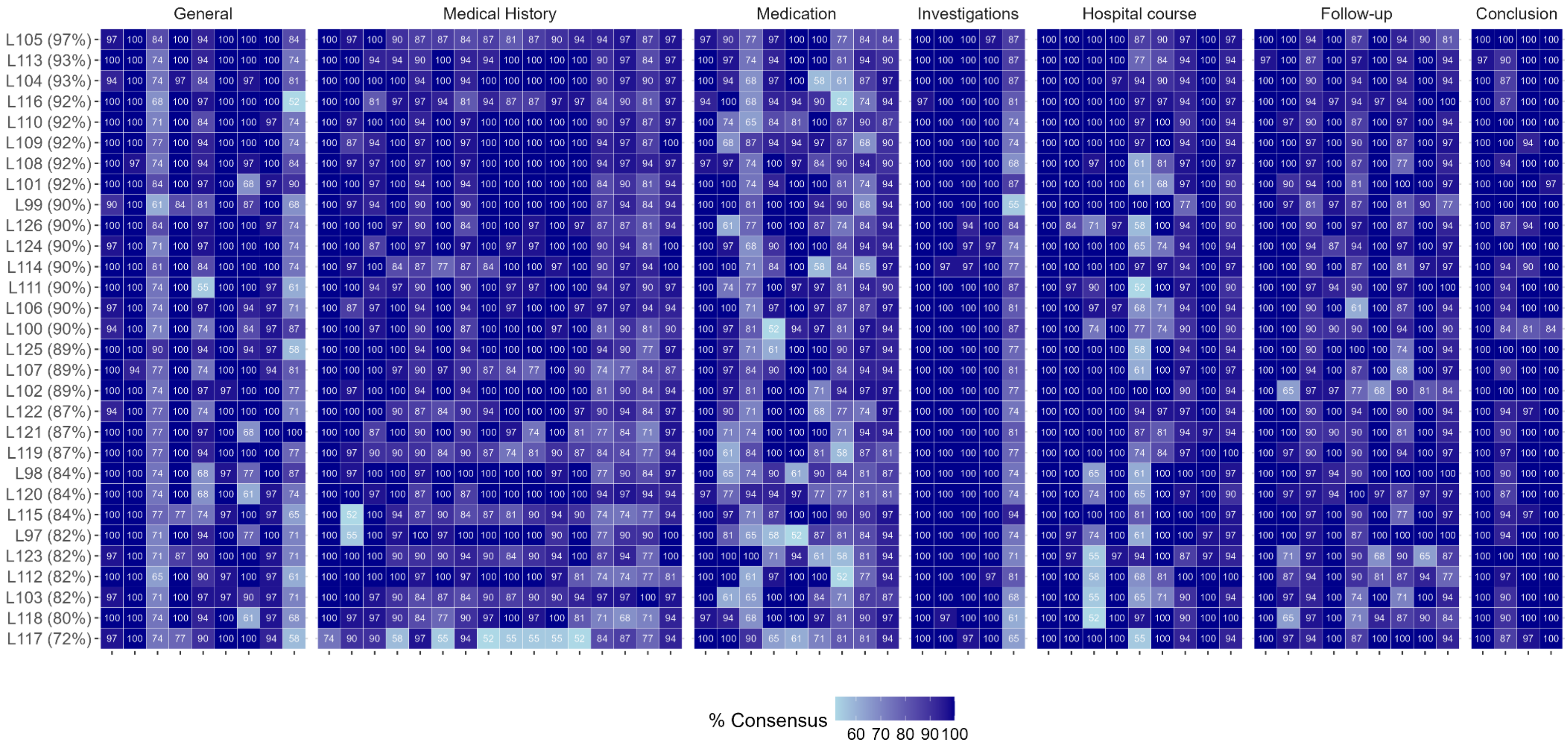

Supplement: Multimedia Appendix 7 [file medinform-v14-e90374-s007.pdf]
