# Supplementary material for: Expert Evaluation and Consensus on GPT-4o Summaries of Clinical Letters: Validation and Results of the Framework and Implementation of AI Tools Project
Source: JMIR Med Inform. 2026 May 11;14:e90374. doi: 10.2196/90374 (PMC13160486; doi:10.2196/90374)

Appendix 8 – Interrater Agreement

Figure 8.1 Pairwise Cohen's Kappa Agreement Matrix

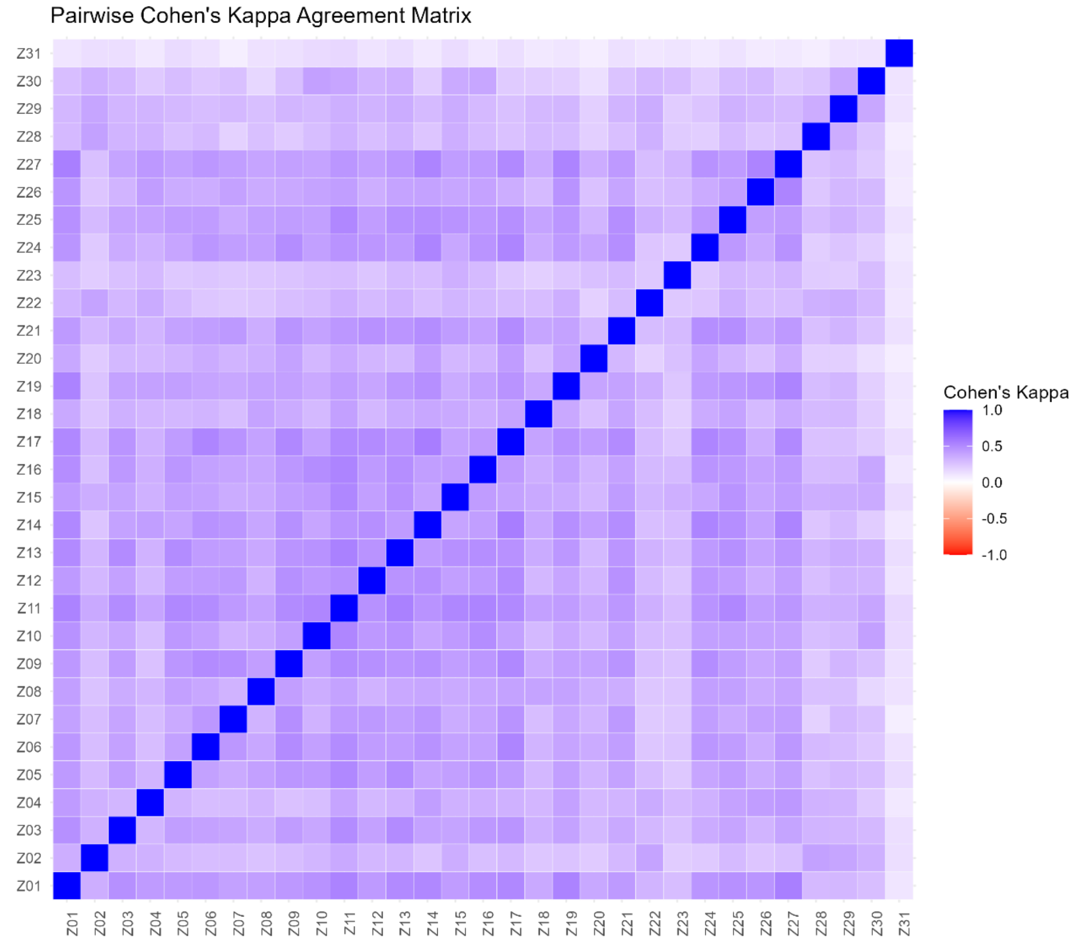

UZ GENT
UNIVERSITEIT GENT
Universitair Ziekenhuis Gent
C. Heymanslaan 10 | B 9000 Gent
www.uzgent.be

Figure 8.2 Proportion of significant comparisons per participant

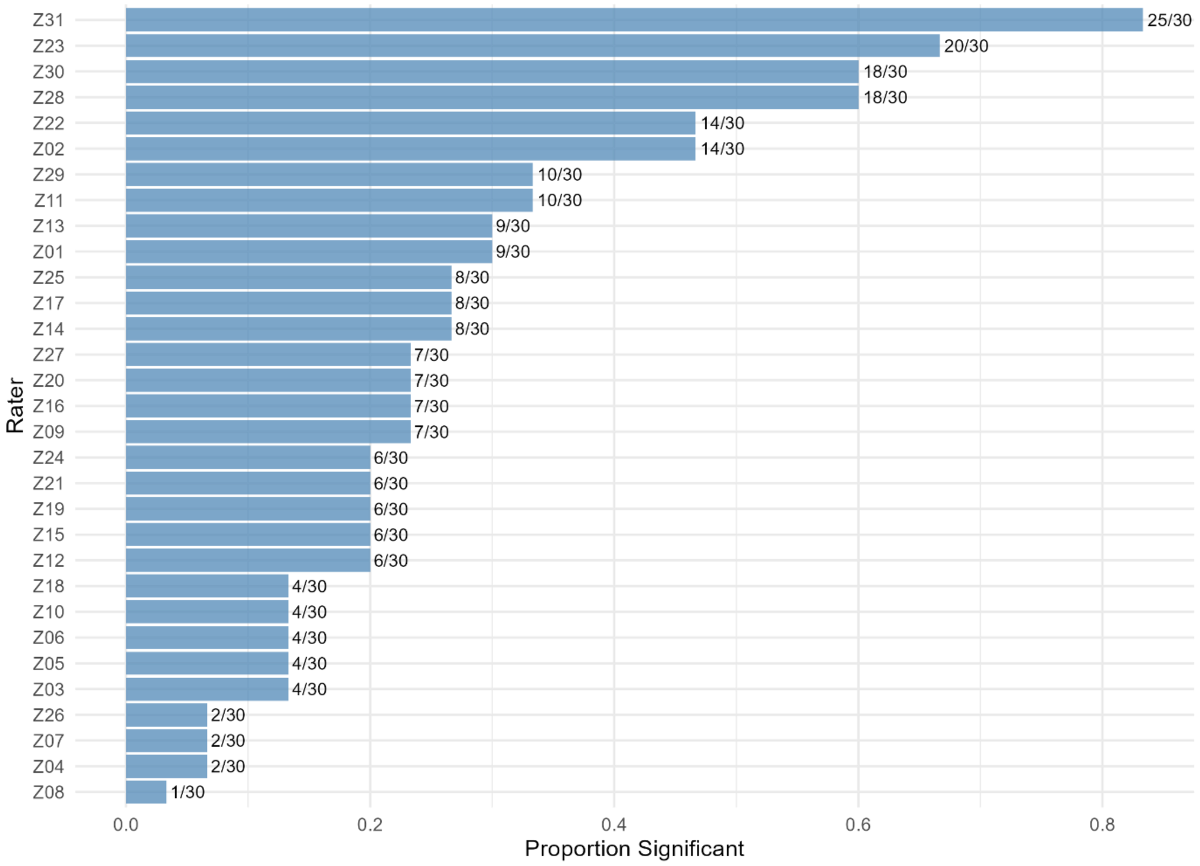

This figure illustrates, for each rater, the proportion of pairwise comparisons in which the rater was involved that were statistically significant. The horizontal bars represent individual raters, ordered from highest to lowest proportion of significant comparisons.

- **Interpretation:** A higher proportion indicates that the rater was more frequently associated with significant differences compared to others. This can suggest that the rater's evaluations differ systematically from those of other raters, potentially signaling bias or distinctive rating patterns.
- **Numbers on bars:** Each bar is annotated with the count of significant comparisons over the total number of comparisons for that rater (e.g., 5/20 means 5 significant out of 20 total).
- **Purpose:** This visualization helps identify raters whose judgments deviate most from the group norm, which is important for assessing consistency and reliability in the rating process.

Figure 8.3 Intraclass Correlation Coefficient with and without outlier

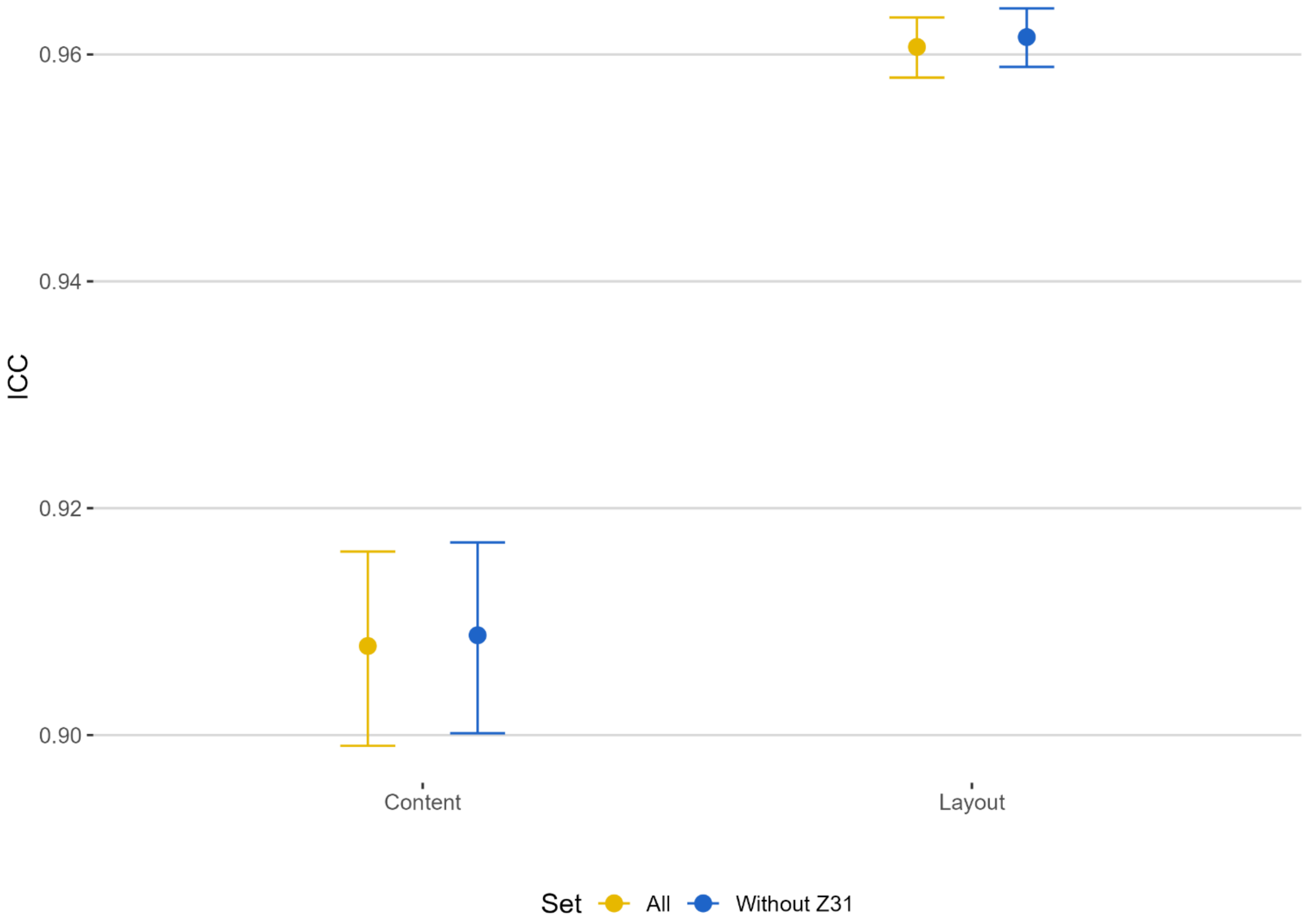

Figure 8.4 Intraclass Correlation Coefficient by category

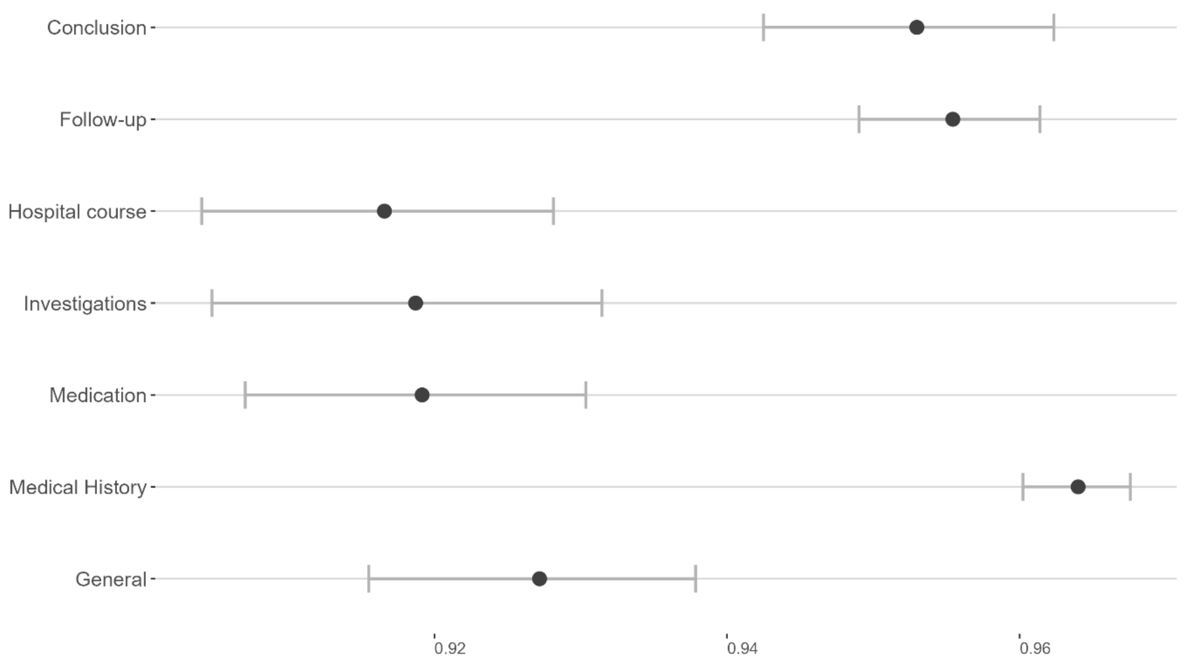

Supplement: Multimedia Appendix 8 [file medinform-v14-e90374-s008.pdf]
